# Annexin A1 exerts renoprotective effects in experimental crescentic glomerulonephritis

**DOI:** 10.3389/fphys.2022.984362

**Published:** 2022-10-12

**Authors:** Robert Labes, Lei Dong, Ralf Mrowka, Sebastian Bachmann, Sibylle von Vietinghoff, Alexander Paliege

**Affiliations:** ^1^ Department of Anatomy, Charité-Universitätsmedizin Berlin, Berlin, Germany; ^2^ Nephrology Department, Tongji Hospital, Tongji College, Huazhong University of Science and Technology, Wuhan, China; ^3^ Klinik für Innere Medizin III, AG Experimentelle Nephrologie, Universitätsklinikum Jena, Jena, Germany; ^4^ Nephrology Section, First Medical Clinic, University Clinic and Rheinische Friedrich-Wilhelms Universität Bonn, Bonn, Germany; ^5^ Division of Nephrology, Department of Internal Medicine III, Technische Universität Dresden, Dresden, Germany; ^6^ Berlin Institute of Health (BIH), Berlin, Germany

**Keywords:** annexin A1 (AnxA1), nephrotoxic serum nephritis, crescentic glomerulonephritis, renal fibrosis, neutrophil granulocyte (PMN), prostaglandin E2 (PGE2), resolution of inflammation, epoxydocosapentaenoic acids (EDPs)

## Abstract

Non-resolving inflammation plays a critical role during the transition from renal injury towards end-stage renal disease. The glucocorticoid-inducible protein annexin A1 has been shown to function as key regulator in the resolution phase of inflammation, but its role in immune-mediated crescentic glomerulonephritis has not been studied so far.

**Methods:** Acute crescentic glomerulonephritis was induced in annexin A1-deficient and wildtype mice using a sheep serum against rat glomerular basement membrane constituents. Animals were sacrificed at d5 and d10 after nephritis induction. Renal leukocyte abundance was studied by immunofluorescence and flow cytometry. Alterations in gene expression were determined by RNA-Seq and gene ontology analysis. Renal levels of eicosanoids and related lipid products were measured using lipid mass spectrometry.

**Results:** Histological analysis revealed an increased number of sclerotic glomeruli and aggravated tubulointerstitial damage in the kidneys of annexin A1-deficient mice compared to the wildtype controls. Flow cytometry analysis confirmed an increased number of CD45^+^ leukocytes and neutrophil granulocytes in the absence of annexin A1. Lipid mass spectrometry showed elevated levels of prostaglandins PGE2 and PGD2 and reduced levels of antiinflammatory epoxydocosapentaenoic acid regioisomers. RNA-Seq with subsequent gene ontology analysis revealed induction of gene products related to leukocyte activation and chemotaxis as well as regulation of cytokine production and secretion.

**Conclusion:** Intrinsic annexin A1 reduces proinflammatory signals and infiltration of neutrophil granulocytes and thereby protects the kidney during crescentic glomerulonephritis. The annexin A1 signaling cascade may therefore provide novel targets for the treatment of inflammatory kidney disease.

## Introduction

Intense inflammation, necrotic destruction of the glomerular tuft and the subsequent formation of crescentic scars (crescentic glomerulonephritis, cGN) are defining features of rapidly progressive glomerulonephritis. The natural course of this condition is characterized by an acute onset of renal dysfunction with marked glomerular hematuria, proteinuria, and a rapid loss of excretory kidney function. Despite the utilization of harsh immunosuppressive treatment, cGN frequently causes chronic, progressive kidney injury and remains a common cause of end stage renal disease (Saran et al., 2019; Trimarchi, 2021), therefore highlighting the need for novel renoprotective therapeutic strategies (Kidney Disease: Improving Global Outcomes (KDIGO) Glomerular Diseases Work Group, 2021).

Previous studies have identified a variety of intrinsic mediators which are activated during the resolution phase of acute inflammation and exert antiinflammatory effects (Ortega-Gomez et al., 2013). The calcium- and phospholipid-binding protein annexin A1 (AnxA1) inhibits leukocyte recruitment and adhesion to endothelial cells (Brancaleone et al., 2011), promotes apoptosis of neutrophils in the inflamed tissue (Vago et al., 2012) and suppresses the synthesis of essential proinflammatory cytokines (Pupjalis et al., 2011; Sugimoto et al., 2016) and eicosanoid species (Herbert et al., 2007; Wang et al., 2011; Seidel et al., 2012). AnxA1 was therefore proposed to play a central role in the resolution of inflammation (Gobbetti and Cooray, 2016). In addition, AnxA1 can be released by damaged and dying cells to act as a “find me” and “eat me” signal for phagocytes. Thereby, AnxA1 may contribute to the effective clearance of apoptotic and necrotic cells and thus help to avoid secondary damage and autoimmunity (Scannell et al., 2007; Blume et al., 2012). AnxA1 is abundantly expressed in the kidney but may also be introduced to the site of damage by infiltrating leukocytes and fibroblasts (McKanna et al., 1992; Dreier et al., 1998; Seidel et al., 2012; Neymeyer et al., 2015; Wang et al., 2022). Evidence for a significant contribution of leukocyte species to the overall tissue abundance of AnxA1 in the diseased kidney is also provided by recent single cell RNA-seq data which show a high abundance of AnxA1 mRNA in fibroblasts, monocytes and other leukocyte subspecies (https://www.kpmp.org).

Renoprotective effects of AnxA1 have been demonstrated for a variety of not primarily immune-mediated conditions including diabetic nephropathy (Wu et al., 2021), ischemia/reperfusion injury (Facio et al., 2011; Suliman et al., 2021) and calcineurin inhibitor toxicity (Araujo et al., 2010; Araujo et al., 2012). Protective mechanisms include antiinflammatory and antifibrotic effects *via* formyl peptide receptor 2 signaling, alteration of MAP kinase signaling and inhibition of TGF-β and NF-kB (Neymeyer et al., 2015; Purvis et al., 2018; Wu et al., 2021).

The regulation of AnxA1 during the course of acute cGN and its effects on renal inflammation and fibrosis in this setting have not been studied. Using the nephrotoxic serum nephritis model of cGN in wildtype (WT) and AnxA1-deficient mice, we here show that AnxA1 protein abundance is increased at d10 after nephritis induction in WT mice. Localization studies revealed an accumulation of AnxA1 immunoreactive protein in regions with damaged glomeruli and nephron segments, corresponding to inflammatory infiltrates and fibrotic scar tissue. At this point in time, proteinuria, morphological damage markers, leukocyte infiltration, expression of proinflammatory cytokines and fibrotic markers were all increased in AnxA1-deficient mice compared to the WT mice. Our data demonstrate a protective role of intrinsic AnxA1 in this model for acute cGN and provide a rational for further studies exploring AnxA1-based pharmacological treatment strategies in inflammatory diseases of the kidney.

## Materials and methods

### Animal studies and tissue preservation

Animal studies were approved by the Berlin Council on Animal Care (permission no. G0251/14) and performed according the NIH Guide for the Care and Use of Laboratory Animals. AnxA1-deficient mice were originally generated by (Hannon et al., 2003) and bred in the Charité animal facility maintaining a C57Bl/6 genetic background. AnxA1-deficient and WT mice were derived from heterozygous breeder pairs and maintained in homozygous colonies under identical environmental conditions.

cGN was induced by intraperitoneal injection of 400 µl of a sheep serum raised against rat glomerular basement membrane constituents (PTX-001S; PROBETEX, San Antonio, United States). The dose was chosen based on published data (Moll et al., 2018; Ougaard et al., 2018) and on preliminary studies in which this dose showed a robust nephritic response without excessive mortality of AnxA1-deficient mice. The same batch of serum was used throughout the study to avoid variations in composition and activity. Control mice for both genotypes received an injection with normal sheep serum (Dianova, Hamburg, Germany). 18 h urine samples were collected on day 4 and 9, and the experiment was terminated at d5 or d10 after nephritis induction.

Kidneys for morphological and immunofluorescence analysis were perfusion-fixed *via* the abdominal aorta using 3% paraformaldehyde in PBS and processed for paraffin or cryostat sectioning as previously described (Paliege et al., 2012). Kidneys for biochemical analysis and flow cytometry were flushed with phosphate buffer (4.3 g/l NaH2PO4 and 14.8 g/l Na2HPO4) (Andrews and Coffey, 1984) to remove blood cells and plasma constituents and either processed immediately for flow cytometry or snap frozen in liquid nitrogen and stored at -80°C.

### Laboratory parameters

Urinary creatinine and albumin concentrations were measured in the Clinical Chemistry Department at the University Hospital Dresden using standard procedures.

### Quantitative real time PCR

Generation of cDNA and subsequent PCR analysis were performed following established methodology (Seidel et al., 2012). In brief, total mRNA from whole kidneys was extracted using the TRIzol™ chloroform extraction method (5Prime, Hamburg, Germany). cDNA was generated by reverse transcription using a kit (Bioline, Luckenwalde, Germany). Expression levels of AnxA1 and Col1A1 were assessed using TaqMan® real time RT-PCR with GAPDH serving as loading control. Probes (AnxA1 #4331182, Col1A1 #4331182, murine GAPDH 4352339E-1108037), and master mix (#4369016) were purchased from Applied Biosystems™ (Darmstadt, Germany). Data were analyzed according to the 2^−∆∆CT^ method, and mRNA levels expressed as x-fold of control (Livak and Schmittgen, 2001).

### Histology and morphometric analysis

Paraffin sections (4 µm) were deparaffinized in Xylol and rehydrated in ethanol and PBS. Periodic acid-Schiff (PAS) staining and Sirius Red staining were performed using established methodology (Junqueira et al., 1979). Stained sections were imaged using a Leica DMRB light microscope equipped with a Zeiss AxioCam MRc digital camera and AxioVision software (Zeiss, Jena, Germany). Quantification of glomerular damage was performed in a blinded manner by counting glomeruli displaying signs of necrosis in the glomerular tuft, crescent formation or global sclerosis. Numbers of glomeruli with crescents and global sclerosis were added and normalized to the total number of glomeruli in the section. Tubulointerstitial damage was determined on blinded sections by visual assessment of the area with interstitial fibrosis and tubular atrophy (Farris et al., 2011). Quantification of Sirius Red signal was performed in an automated image analysis approach using the Fiji distribution of ImageJ (Schindelin et al., 2012). To this end, 6 to 8 adjacent 200 x color images from the kidney cortex were acquired using constant illumination and camera settings. Images were converted to red, green and blue. Sirius Red signal was segmented with a color threshold (HUE min = 232, max = 255; saturation min = 132, max = 255; brightness min = 0, max = 255). The resulting black-and-white image was used to calculate the total signal area and to generate a mask for the measurement of integrated signal intensity with built-in Fiji tool kit.

### Immunofluorescence staining

Four-µm paraffin sections were deparaffinized in xylene and rehydrated in ethanol and PBS. Sections were subsequently boiled in 0.1 M citrate buffer to improve antigen presentation. Cryostat sections were incubated with 0.5% Triton™ X-100 (Sigma-Aldrich, Saint Louis, United States) in PBS for 30 min at room temperature to improve antibody penetration. Unspecific protein binding sites were blocked with 5% skim milk in PBS for 30 min at room temperature. Primary antibodies (Supplementary Table S1) were diluted in milk and incubated overnight at 4 °C. Bound antibodies were detected with Cy2-or Cy3-labeled secondary antibodies (Jackson Laboratories, Bar Harbor, United States). Image acquisition was performed with a Zeiss LSM5 Exciter confocal microscope and ZEN imaging software (Zeiss, Jena, Germany).

### Immunoblotting

Tissue samples were homogenized in liquid nitrogen and dissolved in buffer containing 250 mM sucrose, 10 mM triethanolamine, protease inhibitor (cOmplete™; Roche) as previously described (Seidel et al., 2012; Neymeyer et al., 2015). Samples were sonicated and nuclei removed by centrifugation (1,000 ×*g* for 10 min). Postnuclear supernatants were separated on 10% polyacrylamide mini-gels. Proteins were transferred to nitrocellulose membranes. Unspecific binding sites were blocked with 5% skim milk in PBS for 1 h at room temperature. Antibodies were diluted in milk as detailed in Supplementary Table S1 and applied overnight at 4°C. The abundance of α-tubulin was determined in parallel and served as loading control. Detection of bound primary antibodies was performed by incubation with horseradish peroxidase-conjugated secondary antibodies (Agilent Technologies, Santa Clara, United States) diluted in milk. Signals were generated by incubation with ECL solution (Western Blotting Detection Reagents, GE Healthcare). Image acquisition and signal quantification were performed using an Intas ECL ChemoCam Imager HR 3.2/6.0 (Intas Science Imaging Instruments, Göttingen, Germany).

### Flow cytometry

Fresh kidney samples were prepared for enzymatic digestion as previously described (Ge et al., 2013). In brief, kidneys were dissected in digestion buffer containing 450 U/ml collagenase type I, 250 U/ml collagenase type XI, 120 U/ml hyaluronidase type I-s and 120 U/ml DNAse 1 (all Sigma-Aldrich, Saint Louis, United States) and subsequently incubated for 1 h at 37°C and 150 RPM. The tissue suspension was passed through a cell strainer (mesh size = 70 µm; Corning, New York, United States) to produce a single cell suspension. Staining was performed by mixing the cell suspensions with a cocktail containing the appropriate antibodies (Supplementary Table S1). Yellow and near-infrared LIVE/DEAD™ Fixable Dead Cell Stain Kit (Invitrogen, Carlsbad, CA) was used according to the manufacturer’s instructions. Flow cytometry analysis was performed using a Becton-Dickinson FACSCanto™ II flow cytometer (BD Biosciences, Heidelberg, Germany). Cells were gated for live CD45^+^ cells and normalized to the number of total events to determine the leukocyte abundance in relation to all renal cells. Data analysis was performed using FlowJo™ software (TreeStar Inc., Ashland, OR). The gating strategy is depicted in the Supplementary Figures S1–S3.

### *In situ* hybridization

mRNA *in situ* hybridization for collagen 1 was performed using a probe covering nucleotides 2,256–2,468 of rat collagen 1a2 cDNA (NM_053356). Bound RNA was visualized using 4-nitroblue tetrazolium chloride as described previously (Skogstrand et al., 2013). Stained sections were evaluated using the Leica DMRB microscope.

### Quantification of renal eicosanoid levels

Free tissue levels of eicosanoids and related lipid mediators were determined by mass spectrometry as previously described (Boldt et al., 2016). In brief, kidney samples were finely ground in liquid nitrogen and dissolved in a 50/50 vol/vol mixture of water and methanol supplemented with 0.01% butylhydroxytoluol. Samples were subsequently mixed with 10 µl internal standard solution (0.5 μg/ml) and 2 ml SPE-buffer (0.1 mol/l aqueous sodium acetate solution, pH 6). Solid-phase extraction was performed using a Bond-ElutCertify-II-Column (Phenomenex, Torrance, CA) following the instructions of the manufacturer. Eicosanoids were eluted with 2 ml n-hexane/ethylacetate (25/75 vol/vol) with 1% acetic acid. The solvent was evaporated and residues were resuspended in 100 µl methanol/water mixture. Liquid chromatography-mass spectrometry (LC-MS/MS) was performed at the mass spectrometry facility of Lipidomix (Lipidomix, Berlin, Germany). Values were normalized to the protein content of the sample.

### Gene expression analysis

Transcriptome analysis was performed using Illumina Next Generation Sequencing with an Illumina HiSeq 4000 System and HiSeq Cluster Kit v4 and HiSeq SBS Kit v4 according to the manufacturer’s protocol (Illumina Inc. San Diego, United States). Data from 5 mice per group were obtained and evaluated using the web-based Gene SeT AnaLysis Toolkit (WebGestalt) (Wang et al., 2017). All differentially regulated genes with *p* < 0.05 were included in the analysis. Pathway analysis was conducted using the Gene Ontology Biological processes database (Ashburner et al., 2000). The top 20 pathways sorted by the adjusted *p*-value for down- and upregulated genes are shown.

### Statistical analysis

All statistical analyses were performed using GraphPad Prism 5 software (GraphPad Software, Inc., La Jolla, United States). Differences between survival curves were analyzed using Mantel-Cox log-rank test. Outliers were identified using the ROUT method with Q = 1%. Student’s *t*-test was used for comparisons of renal lipid levels at d10 after nephritis induction; ANOVA with Sidak´s post-hoc test was applied for the comparison of multiple groups as indicated. *p* < 0.05 was considered to be statistically significant.

## Results

### AnxA1 biosynthesis is increased during cGN

**FIGURE 1:**
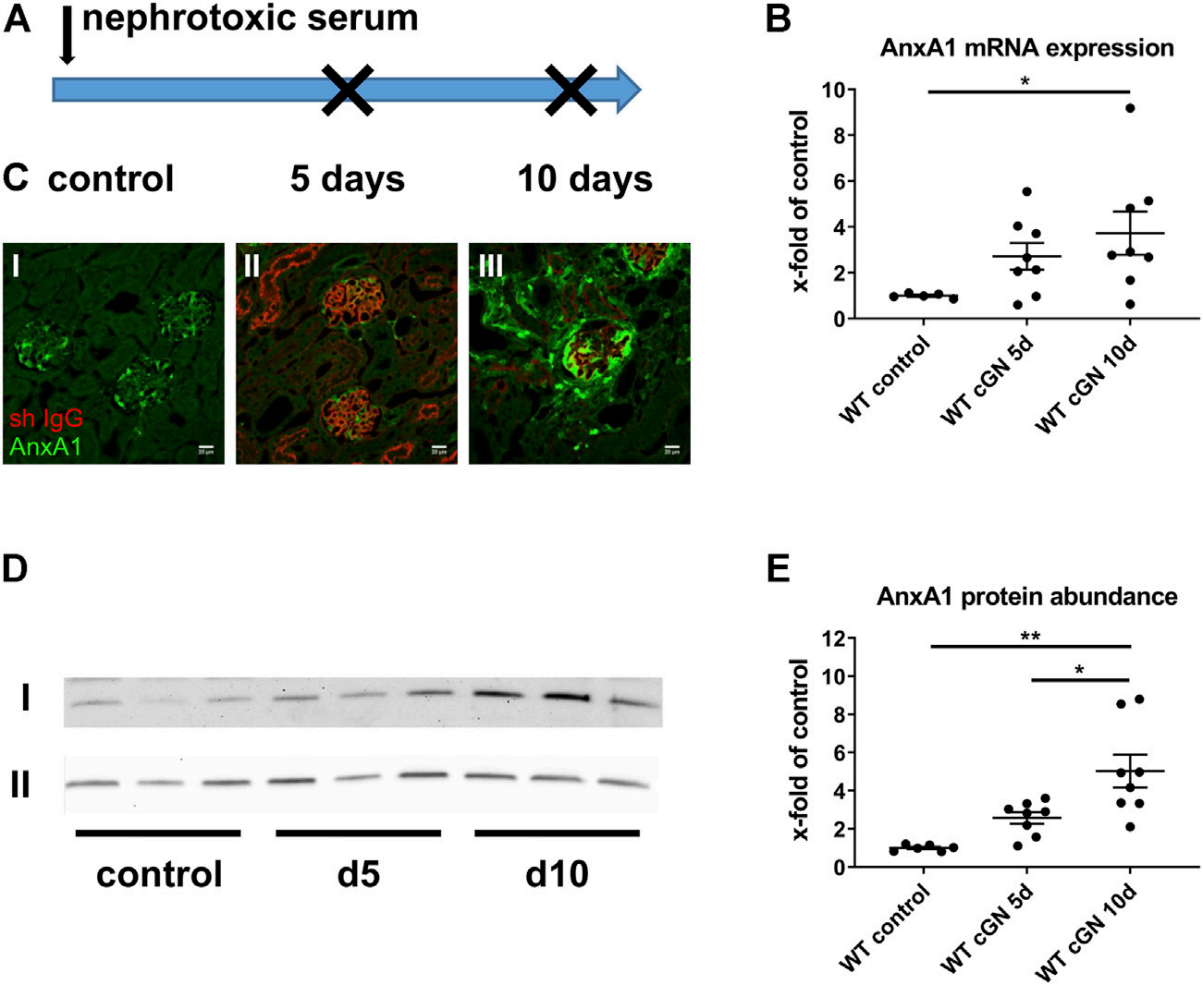
Regulation of annexin A1 during experimental crescentic GN. **(A)** Schematic of experimental setup. Crescentic glomerulonephritis (cGN) was induced at d0 in wildtype (WT) and annexin A1 (AnxA1)-deficient mice by intraperitoneal injection of sheep nephrotoxic serum; animals after injection of normal sheep serum served as controls. Kidneys were harvested at d5 and d10 after cGN induction. **(B)** TaqMan® real time RT-PCR quantification of AnxA1 mRNA abundance in WT mice. **(C)** Representative micrographs showing immunofluorescence staining for AnxA1 (green) and sheep IgG (red) in controls (I) and in mice at 5 (d5; II) and 10 (d10; III) after cGN induction. Scale bar = 20 μm. **(D)** Representative images of Western blots of WT mice treated with control or nephrotoxic serum for 5 (d5) and 10 days (d10). The β-actin content of the tissue homogenates was determined in parallel and served as loading control (lane II). **(E)** Densitometric analysis of the signals in **(D)**. Quantitative data in B and D is shown as x-fold changes in relation to controls. Dots represent measurements from individual mice; horizontal lines indicate mean values and error bars represent the SEM. Statistical significance of changes was calculated using one-way ANOVA and indicated by * for *p* < 0.05 and ** for *p* < 0.01; *n* = 5–8 per group.

Acute cGN was induced in WT and AnxA1-deficient mice by intraperitoneal injection of sheep nephrotoxic serum (Figure 1A). In the first set of experiments, we studied WT mice to determine the effects of nephrotoxic serum injection on renal AnxA1 mRNA expression and protein abundance. Specificity of the AnxA1 antibody was confirmed in AnxA1-deficient mice (Supplementary Figure S4). Quantification of renal AnxA1 mRNA by TaqMan® real time RT-PCR showed a non-significant trend towards higher levels at d5 (272 ± 58% of controls, p = n. s) and significantly elevated levels at d10 (373 ± 94% of controls, *p* < 0.05) after nephritis induction (Figure 1B). Renal localization of AnxA1 signals and deposition of sheep IgG were studied by double-labeling immunofluorescence (Figure 1C). In WT controls, AnxA1 signal was strong in podocytes and weaker in parietal epithelial cells and peritubular fibroblasts, confirming earlier results (McKanna et al., 1992; Seidel et al., 2012)*.* Staining for sheep IgG was absent in the control mice treated with normal sheep serum (Figure 1C, Image I). At d5 after nephrotoxic serum injection, direct immunofluorescence for sheep IgG showed strong linear staining along the glomerular basement membrane, while peritubular capillaries were weakly positive. Immunoreactive signal was further detected in proximal tubular cells, likely representing IgG reabsorbed from the tubular fluid. The glomerular AnxA1 signal was greatly reduced, whereas the tubulointerstitial signal was unchanged (Figure 1C, Image II). At d10, glomerular staining for sheep IgG had decreased which may reflect partial clearance of the antigen. By contrast, AnxA1 immunoreactive signals in the glomeruli and the tubulointerstitium were now markedly increased (Figure 1C, Image III). Immunoblotting for AnxA1 revealed a non-significant trend towards higher protein levels at d5 (257 ± 31% of controls, p = n. s.**)** and, more so, at d10 (503 ± 86% of controls, *p* < 0.01; 196 ± 34% of d5, *p* < 0.05) which agreed with the mRNA levels. As expected, AnxA1 KO mice showed no immunofluorescence signal, thus confirming the specificity of the antibody (Supplementary Figure S4).

In summary, these data show a time-dependent increase of renal AnxA1 abundance during the course of experimental cGN.

### AnxA1 deficiency has no effect on survival but increases proteinuria

**FIGURE 2:**
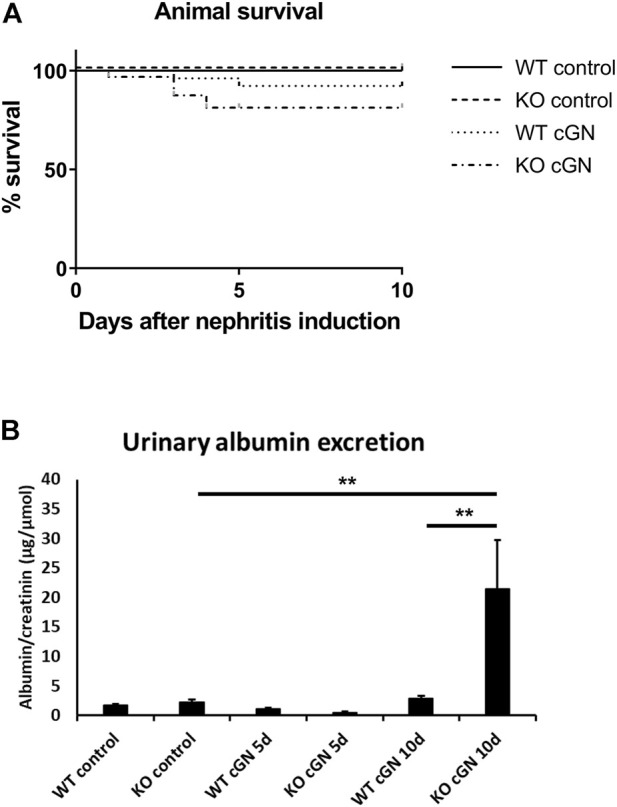
Survival and proteinuria. **(A)** Kaplan-Meier curves showing survival of wildtype (WT) and annexin A1-deficient (KO) mice after induction of crescentic glomerulonephritis (cGN) or after injection of normal sheep serum (control). **(B)** Quantification of urinary albumin-to-creatinine ratio in control mice and at d5 and d10 after cGN induction. Statistical analysis of differences between survival curves was performed using Mantel-Cox log-rank test; proteinuria data was analyzed using two-way ANOVA; *n* = 5–8 per group.

Next, we determined the effect of AnxA1 deficiency on survival and albuminuria. All mice treated with the control serum survived until the end of the study. Survival data from the nephrotoxic serum groups showed no significant differences between WT (11%) and AnxA1-deficient mice (19%). In all cases, death occurred between d1 and d5 after nephritis induction (Figure 2A).

Urinary albumin excretion was determined in 18 h-collection samples and normalized to creatinine concentration. Control mice and mice at d5 after nephritis induction showed low levels of albuminuria, irrespective of genotype. At d10, albuminuria showed a marked 6.6-fold increase in AnxA1-deficient compared to WT mice (+659% ± 288%, *p* < 0.01; Figure 2B).

### Deficiency of AnxA1 aggravates renal damage

**FIGURE 3:**
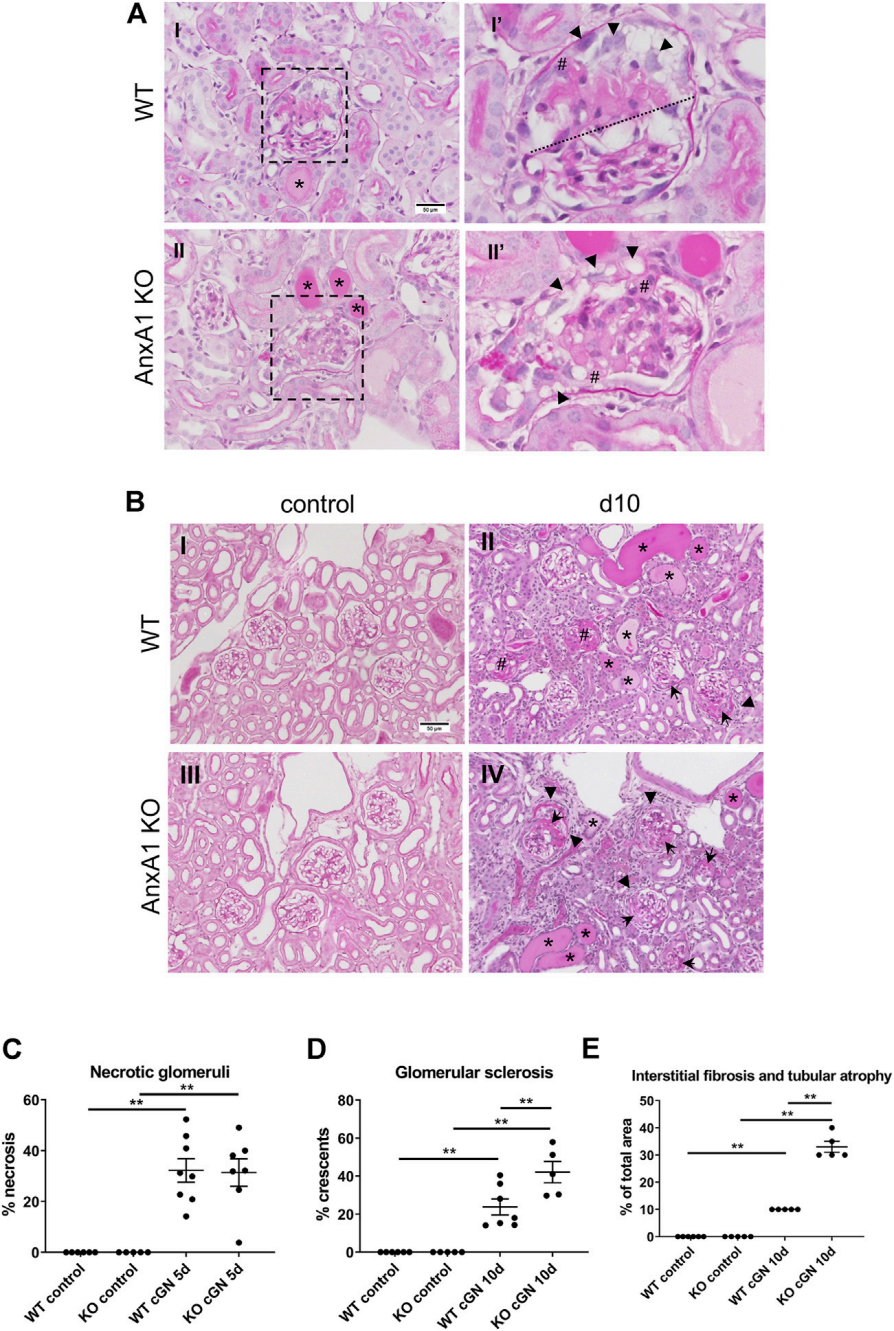
Deficiency of annexin A1 has no effect at d5 but aggravates renal damage at d10. **(A)** Representative micrographs of PAS-stained kidney sections from wildtype (WT; I,I′) and annexin A1 (AnxA1)-deficient mice (KO; II,II′) at d5 after induction of crescentic glomerulonephritis (cGN). Low power micrographs (I,II) show glomeruli with signs of necrosis, tubular profiles filled with protein casts (*) and mild focal interstitial expansion. Magnification of the glomerular profiles boxed in I and II (I′, II′) show segmental (above dotted line in I′) and global necrosis (II′) as well as several tip lesions with attachment of the glomerular tuft to the Bowman capsule (#). Parietal epithelial cells show typical signs of activation with enlarged nuclei and polygonal cell bodies (arrowheads in I′ and II′). Scale bar = 50 µm in I + II. **(B)** Representative micrographs of PAS-stained kidney sections from wildtype (WT; I,II) and annexin A1 (AnxA1)-deficient mice (KO; III,IV) at d10 after injection of normal sheep serum (control; I,III) or induction of cGN (II,IV). Control mice of both genotypes show normal renal morphology. At d10 after cGN induction, glomeruli in mice of both genotypes show morphological hallmarks of extracapillary proliferative glomerulonephritis with crescent formation (arrowheads), global sclerosis (#), and periglomerular fibrosis (arrows). The interstitial space is expanded with an increased number of leukocytes and fibroblasts. Numerous tubular profiles are filled with protein casts (*). Scale bar = 50 µm in I-IV. **(C)** Quantification of glomerular profiles with morphological signs of necrosis at d5 after cGN induction. **(D)** Quantification of glomerular profiles with crescents or global sclerosis at d10 after cGN induction. **(E)** Quantification of areas with tubular atrophy and interstitial fibrosis at d10 after cGN induction. Dots represent values from individual animals; horizontal lines indicate mean values and error bars represent the SEM. Statistical significance of changes was calculated using one-way ANOVA; *n* = 5–8 per group.

Glomerular necrosis, crescent formation, and extent of tubulointerstitial damage were estimated in PAS-stained paraffin sections. Glomeruli from WT and AnxA1-deficient mice frequently showed segmental or global fibrinoid necrosis along with the formation of tip lesions at d5 after nephritis induction. Parietal epithelial cells were activated displaying enlarged nuclei and polygonal cell bodies. Scattered tubular profiles filled with protein precipitates reflected the proteinuria (Figure 3A). Tubular structure was otherwise normal and the tubulointerstitium showed only mild, focal expansion. Morphology in the control mice was normal. Quantification of necrotic glomeruli at d5 after nephritis induction showed significantly increased numbers in nephritic WT (32.2 ± 4.7%, *p* < 0.01) and AnxA1-deficient mice (31.4 ± 5.4%, *p* < 0.01) without significant differences between both genotypes (Figure 3C). The extent of tubulointerstitial damage, expressed by the estimated area with tubular atrophy and interstitial fibrosis, was also similar between WT and AnxA1-deficient mice. At d10, kidneys showed signs of advanced glomerular damage with crescent formation or global sclerosis. Tubulointerstitial damage was increased as well with focal accumulation of leukocytes and fibroblasts in the periglomerular space and around damaged tubules and blood vessels (Figure 3B). Quantification of glomerular profiles with crescents or global sclerosis revealed a significantly increased number of damaged glomeruli in the nephritic WT (23.8 ± 4.2%, *p* < 0.01) and, more severely, in AnxA1-deficient mice (42.1 ± 5.6%, *p* < 0.01). The difference between the nephritic WT and AnxA1-deficient mice was statistically significant (177 ± 24%, *p* < 0.01; Figure 3D).

The tubulointerstitial damage was increased in parallel to the glomerular damage in nephritic WT (10 ± 0%, *p* < 0.01) and AnxA1-deficient mice (33 ± 2%, *p* < 0.01). Again, damage was more severe in the AnxA1-deficient mice (330 ± 20% of WT, *p* < 0.01; Figure 3E).

In summary, these results show a marked aggravation of renal damage in the AnxA1-deficient mice 10 days after nephritis induction.

### AnxA1 deficiency aggravates renal inflammation

**FIGURE 4:**
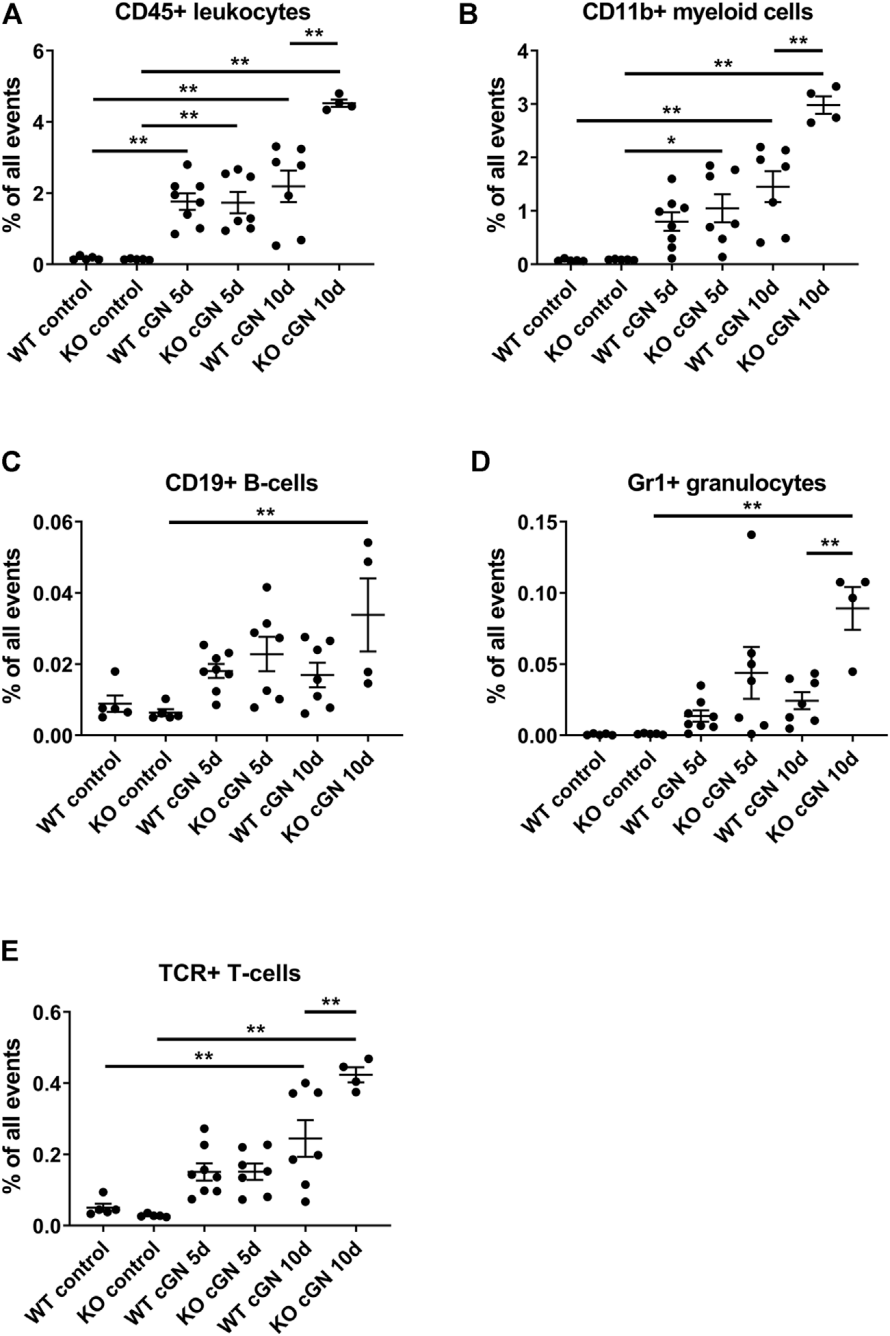
Deficiency of annexin A1 aggravates renal inflammation Flow cytometry quantification of renal CD45^+^ leukocytes **(A)**, CD11b^+^ myeloid cells **(B)**, CD19^+^ B-lymphocytes **(C),** neutrophils defined as live CD45^+^/GR1^HIGH^ cells **(D)** and T-lymphocytes defined as live CD45+/β-TCR^+^ cells **(E)** in wildtype (WT) and annexin A1 (AnxA1)-deficient mice (KO) at d5 and d10 after injection of normal sheep serum (control) or induction of crescentic glomerulonephritis (cGN). Dots represent values from individual animals; horizontal lines indicate mean values and error bars represent the SEM. Statistical significance of changes was calculated using one-way ANOVA; *p < 0.05; **p < 0.01; *n* = 5–8 per group.

To determine the effect of AnxA1 on leukocyte infiltration during cGN, we performed flow cytometry of total kidney single cell suspensions. Accumulation of PMN, macrophages, and T-lymphocytes was further studied using immunofluorescence. Injection of nephrotoxic serum caused renal infiltration of CD45^+^ leukocytes in mice of both genotypes. At d5, numbers were increased 10-fold in WT (1,006 ± 132%, *p* < 0.01) and 12-fold in the AnxA1-deficient mice (1,207 ± 206%, *p* < 0.01); changes were not statistically different between genotypes. At d10, numbers of CD45^+^ cells were increased 12.5-fold in WT (1,248 ± 253%, *p* < 0.01) and 31.5-fold (3,149 ± 687%, *p* < 0.01) in the AnxA1-deficient mice, and changes were significantly different between genotypes (206 ± 4.5%, *p* < 0.01; Figure 4A).

Quantification of CD11b^+^ myeloid cells at d5 revealed a numerical increase in WT (1,038 ± 224%, p = n.s.) and a significant, 11.6-fold increase in the AnxA1-deficient mice (1,164 ± 291%, *p* < 0.05). There was no statistical difference between the genotypes. At d10, the number of CD11b^+^ cells was increased 19-fold in WT (1886 ± 375%, *p* < 0.01) and 33-fold (3,310 ± 185%, *p* < 0.01) in the AnxA1-deficient mice. The difference between the genotypes at d10 was statistically significant (205 ± 11%, *p* < 0.01; Figure 4B).

**FIGURE 5:**
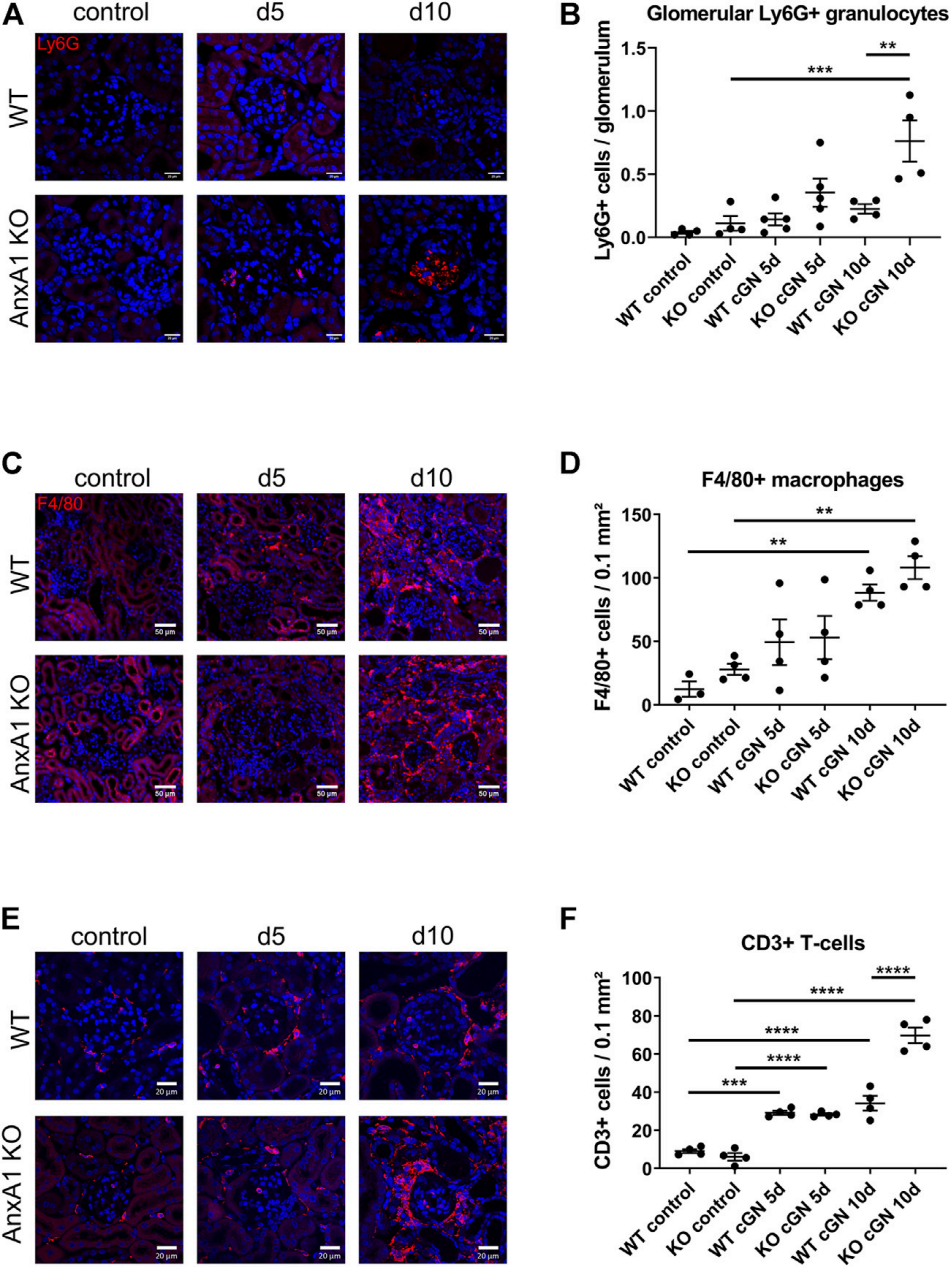
Deficiency of annexin A1 increases the renal abundance of neutrophils, T-lymphocytes and macrophages. **(A)** Representative images of kidney sections from control wildtype (WT) and annexin A1 (AnxA1)-deficient mice (KO) and from mice at d5 and d10 after injection of normal sheep serum (control) or induction of crescentic glomerulonephritis (cGN). Sections were stained for Ly6G as marker for neutrophil granulocytes (red). **(B)** Quantification of glomerular granulocytes shows an increased abundance of Ly6G-positive cells in KO mice at d10 after cGN induction as compared to the nephritic WT mice. **(C)** Representative images of kidney sections from control WT and KO mice and from animals at d5 and d10 after cGN induction. Sections were stained for F4/80 as marker for macrophages (red). F4/80 positive cells accumulate in the renal interstitium of WT and KO animals at d10 after cGN induction. **(D)** Quantification of renal macrophages shows an increased abundance of F4/80 positive cells at d10 after cGN induction without difference between the genotypes. **(E)** Representative images of kidney sections from control WT and KO mice and from animals at d5 and d10 after cGN induction. Sections were stained for CD3 as marker for T-lymphocytes (red). **(F)** Quantification of renal T-lymphocytes shows a significantly increased abundance of CD3-positive cells in the AnxA1-deficient mice at d10 after cGN induction as compared to the nephritic WT mice. Scale bar = 20 μm in A,E and 50 µm in C, blue signal marks DAPI-stained nuclei. Dots in B,D, and F represent values from individual mice; horizontal lines indicate mean values and error bars represent the SEM. Statistical significance of changes was calculated using one-way ANOVA; *p < 0.05; **p < 0.01; ***p < 0.001; *n* = 3–5 per group.

Renal CD19^+^ B-lymphocytes showed a marked, 5-fold increase (529 ± 160%, *p* < 0.01) in the AnxA1-deficient mice at d10 after nephritis induction, despite inter-animal variations, as compared to AnxA1-deficient control mice. Changes in the abundance of CD19^+^ cells in nephritic WT mice were not significant (Figure 5C).

Analysis of GR1^+^ neutrophils revealed a numerical increase at d5 and a significant 9-fold increase at d10 after nephritis induction in AnxA1-deficient mice as compared to the AnxA1-deficient controls (8,804 ± 1,481%, *p* < 0.01). In contrast, renal abundance of GR1^+^ neutrophils was not significantly altered in the WT mice. At d10, renal abundance of Gr1^+^ neutrophils was significantly higher in the AnxA1-deficient mice as compared to the nephritic WT mice (367 ± 62%, *p* < 0.01; Figure 4D).

Quantification of TCR^+^ lymphocyte abundance revealed a numerical increase of positive cells at d5 after nephritis induction without significant genotype-dependent differences. At d10, the number of positive cells was increased 4.8-fold in WT (480 ± 101%, *p* < 0.01) and 15-fold in the AnxA1-defcient mice (1,495 ± 74%, *p* < 0.01). Now the difference between the genotypes was significant (173 ± 9%, *p* < 0.01; Figure 4E).

To localize renal neutrophils, macrophages and T-lymphocytes during nephrotoxic serum nephritis, we performed immunofluorescence staining for Ly6G, F4/80, and, CD3, respectively (Figure 5). Glomeruli of control animals of both genotypes and nephritic WT mice showed only few glomerular profiles with Ly6G^+^ cells without significant differences between the two time points. In contrast, glomeruli in the AnxA1-deficient mice displayed large accumulations of Ly6G^+^ cells at d5 and d10 after nephritis induction. Quantification of glomerular Ly6G^+^ cells in AnxA1-deficient mice revealed a non-significant increase at d5 (320 ± 100%, p = n.s) and a significant 7-fold increase at d10 (688 ± 148%, *p* < 0.001). Comparison of both genotypes at this time point revealed significantly higher numbers of glomerular Ly6G^+^ cells in the AnxA1-deficient mice (338 ± 72%, *p* < 0.01; Figure 5B). Localization studies for F4/80^+^ macrophages in the control mice revealed only very few scattered immunoreactive cells throughout the tubulointerstitium and the perivascular region. Quantification of F4/80^+^ macrophages revealed a non-significant trend towards higher numbers in the AnxA1-deficient mice as compared to WT controls. After nephritis induction, the number of F4/80^+^ immunoreactive cells showed a numerical increase in both, WT and Anxa1-deficient mice at d5 and a further marked increase at d10. Immunoreactive cells were predominantly localized in the tubulointerstitium of the damaged areas whereas glomeruli were generally devoid of F4/80^+^ immunoreactive cells. Quantification of positive cells at d10 after nephritis induction showed a significant 7-fold increase in WT (713 ± 52%, *p* < 0.01) and a 3.8-fold increase in AnxA1-deficient mice (387 ± 32%, *p* < 0.01) relative to the respective control animals (Figure 5D). However, there was no significant difference between the genotypes for both time points.

CD3 immunofluorescence staining in control animals of both genotypes showed few scattered cells in glomeruli, periglomerular region and in the tubulointerstitial space. At d5 after nephritis induction, the overall number of CD3 immunoreactive cells was markedly increased with focal accumulation in the periglomerular space of damaged glomeruli. There was no genotype-dependent difference in the quantity and distribution pattern of the cells. At d10 after nephritis induction, numbers of immunoreactive cells were further increased in the wildtype mice and more dramatically in the AnxA1-deficient mice. Again, immunoreactive cells accumulated in the periglomerular region of the damaged glomeruli and in the tubulointerstitial space of the corresponding nephrons (Figure 5E).

Quantification of CD3^+^ cells in AnxA1-deficient mice revealed a non-significant increase at d5 (320 ± 100%, p = n.s) and a significant 7-fold increase at d10 (688 ± 148%, *p* < 0.001). Comparison of both genotypes at this time point revealed significantly higher numbers of glomerular CD3^+^ cells in the AnxA1-deficient mice (338 ± 72%, *p* < 0.01; Figure 5F).

In the aggregate, these data demonstrate a phenotype of severe progressive inflammation in nephritic AnxA1-deficient mice.

### Deficiency of AnxA1 causes coordinated alterations of renal lipid levels at d10 of experimental cGN

**FIGURE 6:**
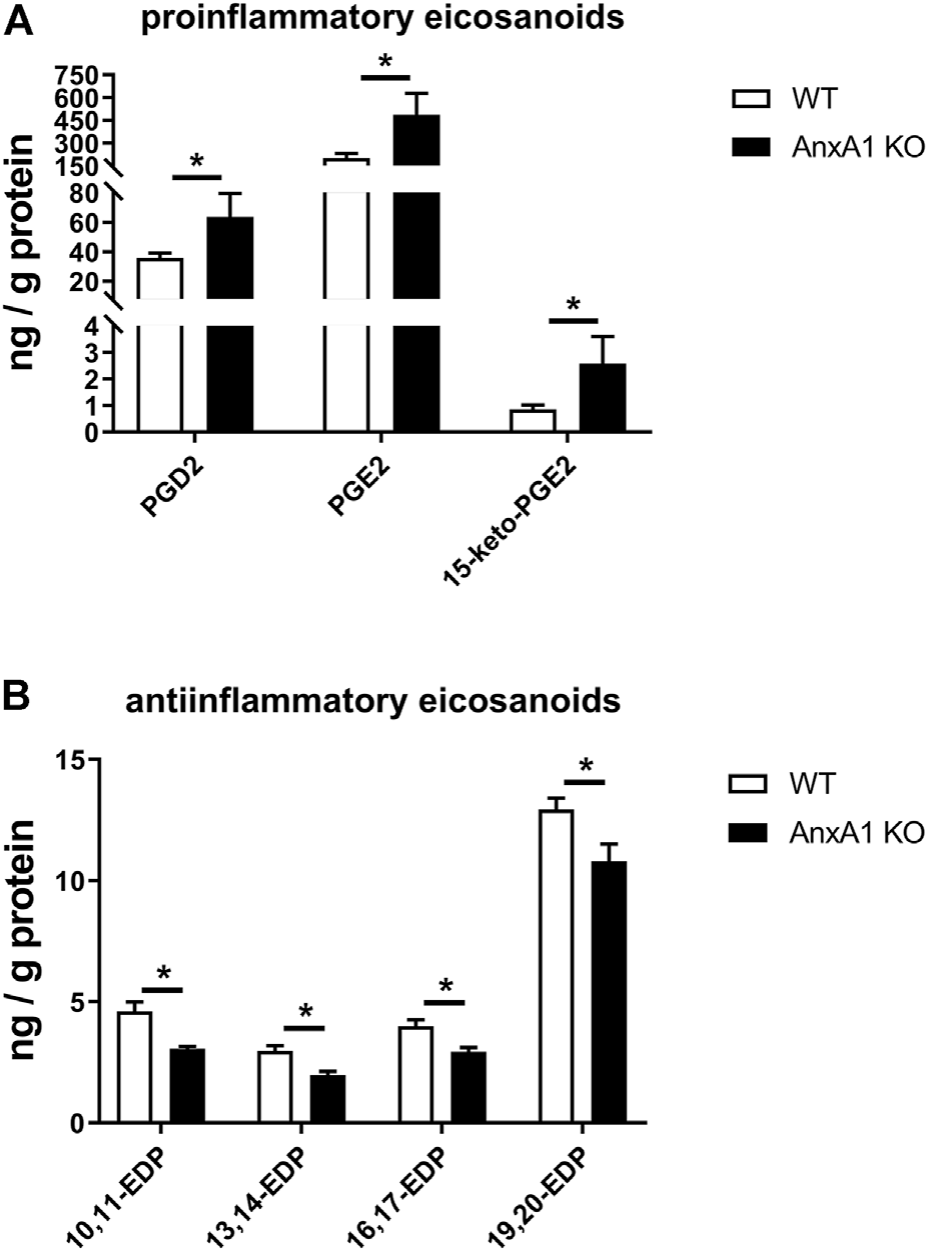
Deficiency of annexin A1 regulates the abundance of proinflammatory and antiinflammatory eicosanoids. **(A)** Lipidomics analysis of proinflammatory renal eicosanoid species in wildtype (WT) and annexin A1 (AnxA1)-deficient mice (AnxA1 KO) at d10 after induction of crescentic glomerulonephritis shows significantly higher tissue levels for prostaglandin D2 (PGD2), prostaglandin E2 (PGE2) and of the principal PGE2 metabolite 15-keto-PGE2 in the AnxA1-KO mice as compared to nephritic WT mice. **(B)** Analysis of antiinflammatory epoxydocosapentaenoic acid (EDP) regioisomers shows significantly lower tissue levels for 10,11-EDP, 13,14-EDP, 16,17-EDP, and 19,20-EDP in AnxA1-KO mice at d10 after induction of crescentic glomerulonephritis as compared to nephritic WT mice. Statistical significance of changes was calculated using Student`s t-test; *p < 0.05; *n* = 5 per group.

Renal tissue levels of eicosanoids and related lipid mediators were measured by mass-spectrometry lipidomics analysis in WT and AnxA1-deficient mice at d10 after nephritis induction. AnxA1-deficient mice displayed increased levels of proinflammatory lipid mediators including PGD2 (178 ± 45%; *p* < 0.05), PGE2 (240 ± 71%; *p* < 0.05) and its degradation product 15-keto-PGE2 (300 ± 120%; *p* < 0.05; Figure 6A). Concomitantly, the abundance of several antiinflammatory epoxydocosapentaenoic acid regioisomers was reduced (10,11-EDP: 66 ± 2%, *p* < 0.05; 13,14-EDP: 66 ± 5%, *p* < 0.05; 16,17-EDP: 74 ± 5%, *p* < 0.05; 19,20-EDP: 84 ± 5%, *p* < 0.05; Figure 6B). Despite extensive efforts, we were unable to detect significant amounts of the canonical proresolving lipid mediators lipoxin A4, resolvins, protectins and maresins in the kidney samples of our experimental animals. A complete list of the lipidomics analysis is presented in Supplementary Table S2.

### Deficiency of AnxA1 causes broad alterations of the renal transcriptome

To assess the influence of AnxA1 on the renal transcriptome in the setting of nephrotoxic serum nephritis, we performed NGS followed by pathway analysis on WT and AnxA1-deficient mice at d10 after nephritis induction. AnxA1 deficiency resulted in differential expression of 1937 genes with downregulation of 1,027 and upregulation of 910 gene products. Pathway analysis of downregulated genes revealed enrichment in pathways relevant for epithelial cell metabolism and function (Table 1). Upregulated genes were enriched in pathways related to leukocyte activation, regulation of cytokine production and secretion as well as leukocyte chemotaxis (Table 2). A comprehensive list of the regulated gene products corresponding to the individual pathways are presented in Supplementary Tables S3, S4.

**TABLE 1:** Functional enrichment of negatively regulated genes annexin A1 deficient mice compared to wildtype mice at d10 of experimental crescentic GN. Regulated gene products corresponding to the individual in pathways are listed in Supplementary Table S2.

Pathway name	O	C	E	R	adjP
Cofactor metabolic process	154	488	55.75	2.76	<9.99E-15
Purine-containing compound metabolic process	132	481	54.95	2.40	<9.99E-15
Ribose phosphate metabolic process	131	443	50.61	2.59	<9.99E-15
Mitochondrion organization	125	434	49.58	2.52	<9.99E-15
Generation of precursor metabolites and energy	118	389	44.44	2.66	<9.99E-15
Small molecule catabolic process	107	328	37.47	2.86	<9.99E-15
Nucleoside monophosphate metabolic process	85	279	31.87	2.67	<9.99E-15
Nucleoside triphosphate metabolic process	83	270	30.85	2.69	<9.99E-15
Cellular amino acid metabolic process	85	260	29.70	2.86	<9.99E-15
Sulfur compound metabolic process	78	275	31.42	2.48	6.58E-13
Dicarboxylic acid metabolic process	41	99	11.31	3.63	1.61E-12
NADH dehydrogenase complex assembly	25	42	4.80	5.21	5.80E-12
Pyridine-containing compound metabolic process	51	150	17.14	2.98	1.17E-11
Organic acid biosynthetic process	85	349	39.87	2.13	3.17E-10
Nucleoside bisphosphate metabolic process	38	106	12.11	3.14	2.00E-09
Cellular ketone metabolic process	53	182	20.79	2.55	2.91E-09
Cellular aldehyde metabolic process	29	70	8.00	3.63	6.61E-09
Tricarboxylic acid metabolic process	20	36	4.11	4.86	6.90E-09
Mitochondrial transport	49	166	18.96	2.58	7.70E-09
Organophosphate biosynthetic process	102	484	55.29	1.84	1.57E-08

O, the number of genes in the gene set and also in the category.

C, the number of reference genes in the category.

E, the expected number in the category.

R, ratio of enrichment.

adjP, *p* value adjusted by the multiple test adjustment.

**TABLE 2:** Functional enrichment of positively regulated genes annexin A1 deficient mice compared to wildtype mice at d10 of experimental crescentic GN. Regulated gene products corresponding to the individual in pathways are listed in Supplementary Table S3.

Pathway name	O	C	E	R	adjP
Regulation of leukocyte activation	136	482	56.31	2.42	<9.99E-15
T cell activation	123	458	53.51	2.30	<9.99E-15
Negative regulation of immune system process	125	431	50.35	2.48	<9.99E-15
Positive regulation of cytokine production	118	404	47.20	2.50	<9.99E-15
Adaptive immune response	116	397	46.38	2.50	<9.99E-15
Regulation of cell-cell adhesion	106	369	43.11	2.46	<9.99E-15
Activation of immune response	111	368	42.99	2.58	<9.99E-15
Positive regulation of defense response	99	341	39.84	2.48	<9.99E-15
Leukocyte migration	97	315	36.80	2.64	<9.99E-15
Immune response-regulating signaling pathway	99	310	36.22	2.73	<9.99E-15
Positive regulation of cell activation	89	306	35.75	2.49	<9.99E-15
Leukocyte cell-cell adhesion	91	301	35.17	2.59	<9.99E-15
Cell chemotaxis	84	280	32.71	2.57	<9.99E-15
Regulation of innate immune response	82	267	31.19	2.63	<9.99E-15
Cytokine secretion	71	213	24.88	2.85	<9.99E-15
Myeloid leukocyte activation	70	194	22.67	3.09	<9.99E-15
Cytokine-mediated signaling pathway	97	360	42.06	2.31	3.18E-14
Interleukin-6 production	54	144	16.82	3.21	3.51E-14
Regulation of inflammatory response	88	313	36.57	2.41	4.74E-14
Negative regulation of cell activation	61	180	21.03	2.90	1.08E-13

O, the number of genes in the gene set and also in the category.

C, the number of reference genes in the category.

E, the expected number in the category.

R, ratio of enrichment.

adjP, *p* value adjusted by the multiple test adjustment.

### Deficiency of AnxA1 increases markers of renal fibrosis

**FIGURE 7:**
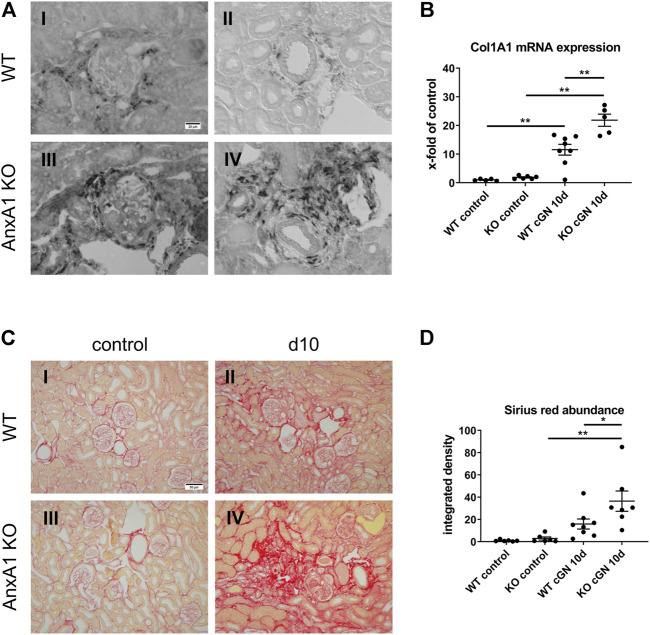
Deficiency of annexin A1 aggravates renal fibrosis. **(A)** Representative images of alpha-1 type 1 collagen (Col1A1) mRNA *in situ* hybridization performed on kidney sections from wildtype (WT; I, II) and annexin A1 (AnxA1)-deficient mice (KO; III,IV) at d10 after injection of normal sheep serum (control) or induction of crescentic glomerulonephritis (cGN). Col1A1 mRNA signal is localized to fibroblasts in the periglomerular region of damaged glomeruli (I,III) and the perivascular space of arcuate arteries (II,IV). **(B)** TaqMan® real time RT-PCR quantification of Col1A1 mRNA abundance in WT and KO mice at d10 after cGN induction. **(C)** Representative images of Sirius Red-stained kidney sections from WT (I,II) and KO (III,IV) mice at d10 after cGN induction in control- (I, III), or cGN mice (II,IV). **(D)** Quantification of renal cortical Sirius Red signal by automated image analysis shows an increased abundance in the KO cGN mice as compared to the nephritic WT mice. Scale bar = 20 μm in A and 50 µm in **(C)**. Dots in B and D represent values from individual mice; horizontal lines indicate mean values and error bars represent the SEM. Statistical significance of changes was calculated using two-way ANOVA; *p < 0.05; **p < 0.01; *n* = 6–8 per group.

Fibrotic scar formation is a consequence of nephron loss during cGN. To assess the extent of renal scarring, we performed *in-situ* hybridization and TaqMan® real time RT-PCR for Col1A1. In addition, the abundance of extracellular collagen fibers was visualized and quantified by Sirius Red Stain with subsequent measurement of the integrated signal intensity. As shown in Figure 7A, Col1A1 mRNA was localized in fibroblasts in the periglomerular region of damaged glomeruli and the perivascular space of arcuate arteries in kidneys of mice at d10 after nephritis induction. Col1A1 mRNA signal abundance was increased in AnxA1-deficient mice compared to their WT counterparts. Quantitative real time RT-PCR revealed a 11.5-fold increase of renal Col1A1 mRNA abundance in WT (1,054 ± 188%, *p* < 0.01) and a 11.7-fold increase in AnxA1-deficient mice (1,169 ± 113%, *p* < 0.01) at d10 after nephritis induction (Figure 7B). Comparison of both genotypes showed a significant higher Col1A1 mRNA expression in kidneys of the AnxA1-deficient mice (189 ± 18%, *p* < 0.01). Under control conditions, Sirius Red-positive extracellular collagen fibers were predominantly located in the vicinity of Bowman’s capsule and in the perivascular space (Figure 7C). 10 days after nephritis induction, Sirius Red-positive collagen fibers accumulated in areas with glomerular and tubulointerstitial damage as well as in the perivascular space of interlobular arteries. At d10 after nephritis induction, quantification of Sirius Red signal demonstrated only a numerical increase in the signal density in WT (165 ± 47%, p = n.s.) and a significant 13-fold increase (1,285 ± 321%, *p* < 0.01) in AnxA1-deficient mice as compared to the respective controls. Comparison of both genotypes at this time point revealed significantly higher Sirius Red signal intensity in the AnxA1-deficient mice (230 ± 57%, *p* < 0.05; Figure 7D).

## Discussion

We hereby show that AnxA1 mRNA expression and protein abundance are increased at d10 after induction of nephrotoxic serum nephritis. Both intrinsic renal cells and infiltrating leukocytes contribute to these elevated levels. Functionally, AnxA1-deficient mice display a phenotype of severe non-resolving inflammation as indicated by elevated tissue levels of leukocytes and proinflammatory mediators including prostaglandins D2 and E2 as well as aggravated glomerular crescent formation, tubulointerstitial damage and severe proteinuria.

Renal expression of AnxA1 and its regulation in kidney disease have been studied in considerable detail (McKanna et al., 1992; Ka et al., 2014; Neymeyer et al., 2015; Wu et al., 2021). In a model of Adriamycin-induced nephropathy, the authors observed a time-dependent increase in glomerular AnxA1 expression with maximum levels in podocytes and parietal epithelial cells at d7 and d14 after induction. Elevated expression at these sites was further reported in biopsies from patients with diabetic kidney disease, cGN and proliferative lupus nephritis (Ka et al., 2014; Wu et al., 2021). Our localization studies recapitulate these findings, thus suggesting that upregulation of AnxA1 in podocytes and parietal epithelial cells may be part of a universal response to glomerular injury. Since AnxA1 is involved in membrane homeostasis (Babiychuk et al., 2011; Potez et al., 2011) and the stabilization of intracellular calcium levels during phases of energy depletion and acidosis (Vais et al., 2020), elevated AnxA1 levels may increase the resilience of these cells against further insults.

A pronounced accumulation of AnxA1 was also observed in the tubulointerstitial space corresponding to inflammatory infiltrates and fibrotic areas. These findings are in line with previous localization studies by us and others (Neymeyer et al., 2015; Wu et al., 2021) and with data from single cell RNA sequencing studies (https://kpmp.org/). Prominent expression of AnxA1 in neutrophils and macrophages underlines the importance of these cells for the generation of pro-resolving signals in the inflamed tissue (Sugimoto et al., 2016).

In a detailed analysis of renal leukocyte species by flow cytometry and immunohistology, we found no difference in the extent of renal leukocyte infiltration between wildtype and AnxA1-deficient mice at d5 after induction of nephrotoxic serum nephritis. In addition, the evaluation of glomerular profiles at this time point revealed a similar damage pattern without significant differences between genotypes. These findings argue against a major protective role of AnxA1 during the initiation phase of nephrotoxic serum nephritis. In contrast, at d10 after nephritis induction, we observed higher numbers of infiltrating leukocytes along with increased glomerular and tubulointerstitial damage in the AnxA1-deficient mice as compared to the WT controls. Analysis of leukocyte populations by flow cytometry and immunofluorescence revealed an increased abundance of lymphocytes, macrophages and neutrophil granulocytes. To further corroborate these findings, we performed RNAseq gene expression analysis which revealed increased mRNA levels of gene products involved in neutrophil activation and chemotaxis as well as macrophage activation. Reduced mRNA levels of gene products involved in metabolic processes likely reflect the loss of transporting nephron epithelia. These findings therefore present hallmark features of non-resolving inflammation and the associated progressive destruction of functional renal tissue.

A central result of our study is the highly increased abundance of neutrophils in the glomeruli and in the tubulointerstitium of AnxA1-deficient mice at d10 after nephritis induction. Typically, neutrophils are among the first leukocytes to arrive in the glomerulus after induction of nephrotoxic serum nephritis (Kuligowski et al., 2006) where they contribute to glomerular damage by generating neutrophil extracellular traps (NET) (Westhorpe et al., 2017) and by initiating the respiratory burst reaction (Suzuki et al., 2003; Xiao et al., 2005; Kurts et al., 2013; Antonelou et al., 2022). Neutrophil recruitment may either occur *via* direct Fcγ receptor/Mac-1-mediated binding of antibodies on the glomerular basement membrane (Suzuki et al., 2003) or in a mechanism depending on platelet-derived P-selectin (Kuligowski et al., 2006). In resolution-competent animals these neutrophils are rapidly cleared during the first 72 h after nephritis induction (Wu et al., 1996; Devi, 2013 #44). In line with this timeframe, we found only few neutrophils in glomeruli of the wildtype mice at d5 and d10 after nephritis induction. Mechanisms of neutrophil clearance in the setting of nephrotoxic serum nephritis-induced cGN have not been determined. However, our finding of increased numbers of neutrophils in the kidneys of AnxA1-deficient mice suggests an essential role for AnxA1 during this process.

Importantly, neutrophils themselves are an essential source for AnxA1 in the inflammatory microenvironment (Vergnolle et al., 1995). In neutrophils, AnxA1 is localized in gelatinase granules and in the cytosol (Perretti et al., 2000) and is released during degranulation and NETosis. After externalization, AnxA1 is subject to proteolytic cleavage by neutrophil elastase and proteinase 3 which results in the release of bioactive, soluble N-terminal protein fragments (Oliani et al., 2001; Rescher et al., 2006; Vong et al., 2007). Both full-length AnxA1 and its fragments have been shown to contribute to the antiinflammatory actions of the protein (Walther et al., 2000; Bode et al., 2019). Multiple effects of AnxA1 on neutrophil function have been described (Sugimoto et al., 2016). AnxA1 reduces neutrophil adhesion to endothelial cells by promoting the shedding of L-selectins and by counteracting the activation and clustering of essential integrins (Solito et al., 2003; Drechsler et al., 2015). In the inflamed tissue, AnxA1 induces neutrophil apoptosis in a calcium and FPR2-dependent mechanism (Solito et al., 2003; Dalli et al., 2013). The apoptotic bodies arising from this process are important mediators of the resolution phase of acute inflammation since they function as decoy receptors for proinflammatory cytokines (Jones et al., 2016). Efferocytosis of neutrophil-derived apoptotic bodies by macrophages induces a pro-resolving phenotype with increased phagocytic activity and expression of antiinflammatory cytokines including IL-10, TGF-β (Sugimoto et al., 2016). In addition, AnxA1 reduces platelet P-selectin levels (Vital et al., 2020) which may be of particular relevance for the suppression of glomerular neutrophil infiltration during nephrotoxic serum nephritis.

Various components of the adaptive immune system have been implicated in the pathogenesis of cGN (Artinger et al., 2017; Chen et al., 2018). In particular, gamma delta T cells and their principal cytokine, interleukin 17, have been shown to be instrumental for neutrophil recruitment and the development of renal injury in nephrotoxic serum-induced cGN (Turner et al., 2012; Disteldorf et al., 2015).

Studies addressing the role of AnxA1 in models of Th17-dependent autoimmune diseases have produced conflicting results with protective effects reported in mouse models of retinal inflammation (Yazid et al., 2015), collagen-induced arthritis, contact dermatitis (Yang et al., 2013) and in patients with multiple sclerosis (Colamatteo et al., 2019). In contrast, in mouse models of allergic airway inflammation and experimental autoimmune encephalomyelitis AnxA1-deficient mice showed decreased Th1/Th17 polarization of T cells and reduced disease activity, suggesting proinflammatory effects of AnxA1 (D'Acquisto et al., 2007; Paschalidis et al., 2009).

In the present study, we observed increased numbers of Th17-expressing T cells at d5 which decreased at d10, thus confirming previous reports regarding the kinetics of Th17 T-cells in the nephrotoxic serum nephritis model (Turner et al., 2012). Unexpectedly, we found no genotype-dependent differences in the number of Th17-expressing T cells for both points in time. Furthermore, RNAseq gene expression analysis showed no genotype-dependent difference in the mRNA levels of IL-17 family members and their putative receptors at d10 after nephritis induction. The mRNA abundance of the principal effector cytokines of TH17 cells, CXCL1, and CXCL5 remained unchanged as well (data not shown). In aggregate, these findings therefore argue against an important contribution of Th17 T-cells to the excessive inflammation and tissue destruction observed in the AnxA1-deficient mice. However, further studies are needed to fully elucidate the effects of AnxA1 on Th17 T-cell generation and function.

Prostaglandins and related lipid mediators control important aspects of kidney function and inflammation in cGN (Takahashi et al., 1990; Kvirkvelia et al., 2013). Since AnxA1 has been shown to inhibit renal prostaglandin synthesis by interfering with phospholipase A2 and cyclooxygenase 2, the two rate-limiting enzymes involved in the generation of PGE2, we measured renal levels of PGE2 and other polyunsaturated fatty acids metabolites. Using an unbiased state-of-the-art lipidomics approach, we found markedly elevated levels of PGE2 and its predominant metabolite 15-keto-PGE2 in the kidney homogenates of the AnxA1-deficient mice at d10 after nephritis induction. This finding is in line with the well-established inhibitory effect of AnxA1 on renal PGE2 synthesis and thus confirms previous studies (Seidel et al., 2012). The functional consequences of these elevated PGE2 levels are not well understood and may involve proinflammatory effects due to increased Cxcl-5 expression (Aringer et al., 2018) on the one hand and tissue-protective and repair-promoting effects on the other (Kvirkvelia et al., 2013). However, since PGE2 has been shown to cause glomerular hyperfiltration in the setting of cGN (Takahashi et al., 1990), we speculate that these hemodynamic effects are also present in our animals. In fact, glomerular hyperfiltration due to PGE2-mediated dilatation of the afferent arteriole may explain the severe proteinuria and excessive glomerular damage in these animals.

An unexpected finding of the lipidomics analysis was the marked reduction of renal epoxydocosapentaenoic acid levels. These compounds are produced from docosahexaenoic acid in a cytochrome P450 epoxygenase-dependent manner and subject to inactivation by soluble epoxide hydrolase (Spector and Kim, 2015). Their biological functions include potent antihypertensive, antiinflammatory, antifibrotic, and antiangiogenetic effects (Spector and Kim, 2015; Sharma et al., 2016; Schunck et al., 2018). Reduced levels of these compounds in the nephritic AnxA1-deficient mice may therefore contribute to the aggravated damage. However, further studies are needed to elucidate the effects of AnxA1 on the metabolism of these compounds and their interaction with AnxA1 on the functional level.

Of note, we were unable to detect relevant levels of the canonical pro-resolving lipid mediators lipoxin A4, resolvin, and maresin, while other metabolites of their parent molecules were readily detectable. Technical reasons may be responsible for this finding. The characterization of SPM biosynthesis has typically relied on analyses of exudates from mouse models of resolving inflammation but measurement of tissue levels of these compounds is not routinely performed. Thus, the relevance of these mediators during the resolution process of cGN remains to be determined.

The final fundamental result of our study is the accelerated development of glomerulosclerosis and tubulointerstitial fibrosis in the nephritic AnxA1-deficient mice. Renal fibrosis is the final common pathway mediating the transition from injury to chronic renal failure irrespective of the underlying disease (Humphreys, 2018; Moeller et al., 2021). Since the excessive deposition of extracellular matrix components and the resulting rarefication of the renal microvasculature cause tissue hypoxia, fibrosis by itself has been suggested to contribute to the development of renal damage.

Numerous studies have demonstrated an antifibrotic effect of AnxA1 in different models of tissue fibrosis (Damazo et al., 2011; Locatelli et al., 2014; Wu et al., 2021; Gadipudi et al., 2022). In addition to its antiinflammatory and tissueprotective activity, AnxA1 directly counteracts the effects of TGF- β on renal fibroblasts (Neymeyer et al., 2015) and blocks the profibrotic effects of NF-kΒ (Wu et al., 2021). These findings suggest that, in addition to the aggravation of renal inflammation, tissue damage and increased proteinuria, lack of AnxA1 may also directly contribute to the accelerated kidney fibrosis observed in the AnxA1-deficient mice. Further studies are needed to elucidate the relative contributions of the different mechanisms to the development of renal fibrosis in cGN.

## Conclusion

In conclusion, we have shown that AnxA1-deficient mice show a severely aggravated course of nephrotoxic serum nephritis with increased glomerular and tubulointerstitial damage as well as proteinuria. Renal damage was accompanied by non-resolving inflammation with elevated expression of pro-inflammatory cytokines and an increased abundance of neutrophil granulocytes and other leukocyte species. These data therefore demonstrate a non-redundant role of AnxA1 during the resolution phase of renal immune-mediated inflammatory disease and provide the rational for the development of AnxA1-based therapeutic strategies for these conditions.

## Data Availability

The datasets presented in this study can be found in online repositories. The names of the repository/repositories and accession number(s) can be found below: https://www.ncbi.nlm.nih.gov/, GSE204873.
