# Supplementary material for: Annexin A1 exerts renoprotective effects in experimental crescentic glomerulonephritis
Source: Front Physiol. 2022 Oct 12;13:984362. doi: 10.3389/fphys.2022.984362 (PMC9605209; doi:10.3389/fphys.2022.984362)
Supplement: Supplementary file 1 [file Table1.DOCX]

**Supplementary Table S1: List of antibodies**

| Antibody | Clone | Host | Technique | Dilution | Cat-No. | Manufacturer |
| --- | --- | --- | --- | --- | --- | --- |
| anti-AnxA1 | polyclonal | rabbit | IF paraffin  WB | 1:500  1:1000 | LS-B6711 | Lifespan Biosciences, Cupertino, USA |
| anti-α-tubulin | B-5-1-2 | mouse | 1:2000 | WB | T5168 | Sigma-Aldrich, Saint Louis, USA |
| anti-Ly6G | 1A8 | rat | IF paraffin | 1:100 | 127601 | Biolegend, San Diego, USA |
| anti-CD3 | polyclonal | rabbit | IF paraffin | 1:100 | A0452 | Agilent/Dako, Santa Clara, USA |
| anti-F4/80 | CI:A3-1 | rat | IF Cryo | 1:100 | MCA497R | BIO-RAD, Richmond, USA |
| anti-CD45 PerCP | 30-F11 | rat | FACS | 1:100 | 561047 | BD Pharmingen, New Jersey, USA |
| anti-CD11b PE | M1/70 | rat | FACS | 1:100 | 101207 | Biolegend, San Diego, USA |
| anti-CD11c PerCP/Cy5.5 | N418 | hamster | FACS | 1:100 | 117327 | Biolegend, San Diego, USA |
| anti-GR1 PE/Cy7 | RB6-8C5 | rat | FACS | 1:100 | 108415 | Biolegend, San Diego, USA |
| anti-CD19 PE/Cy7 | 6D5 | rat | FACS | 1:100 | 115519 | Biolegend, San Diego, USA |
| anti-TCRβ APC/Cy7 | H57-597 | hamster | FACS | 1:100 | 109219 | Biolegend, San Diego, USA |
| anti-TCRβ APC | H57-597 | hamster | FACS | 1:100 | 109211 | Biolegend, San Diego, USA |
| anti-IL-17A PE | TC11-18H10.1 | rat | FACS | 1:100 | 506903 | Biolegend, San Diego, USA |
