# Supplementary figures and images for: Annexin A1 exerts renoprotective effects in experimental crescentic glomerulonephritis
Source: Front Physiol. 2022 Oct 12;13:984362. doi: 10.3389/fphys.2022.984362 (PMC9605209; doi:10.3389/fphys.2022.984362)

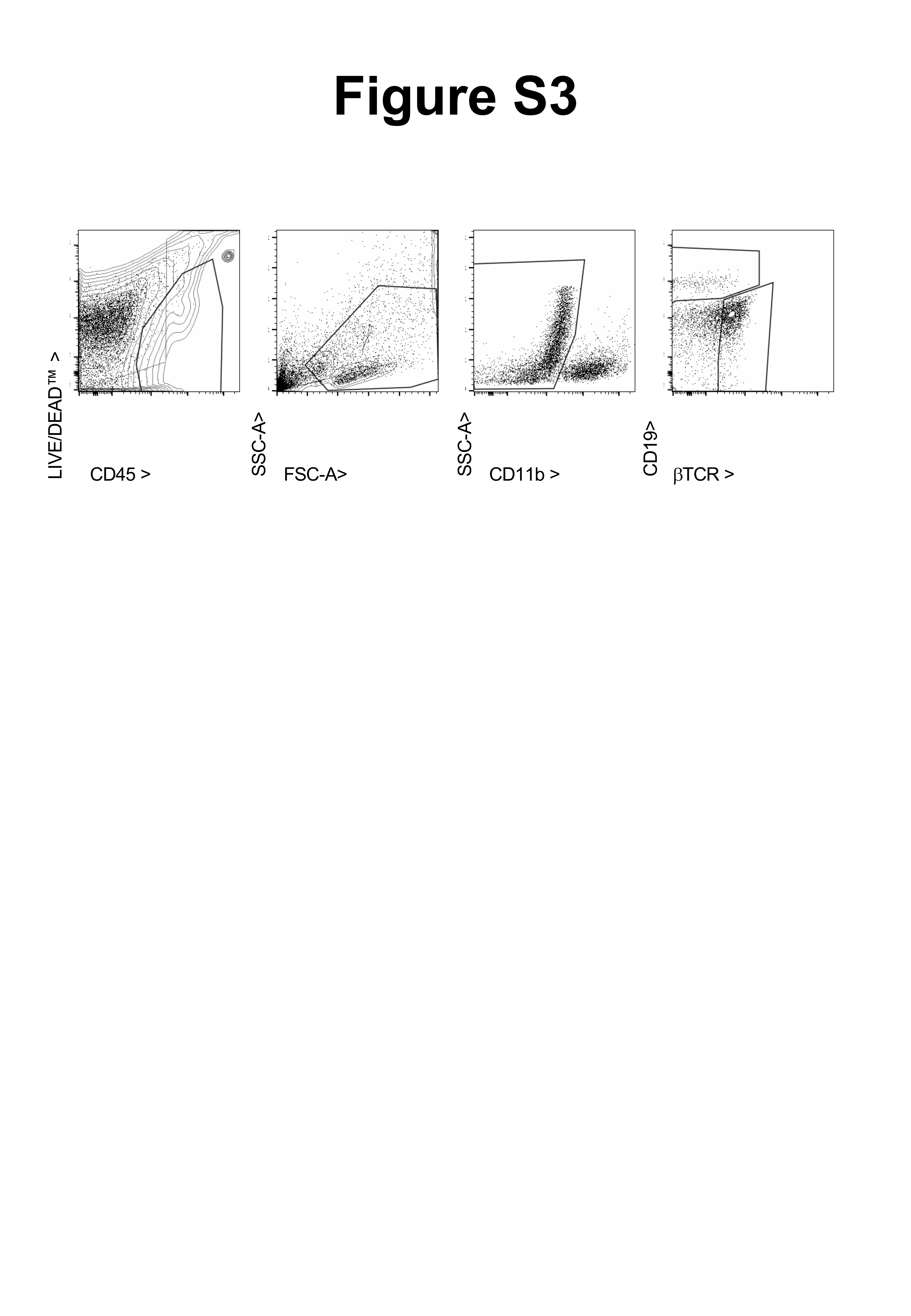

Supplement: Supplementary file 2 [file Image3.TIF]

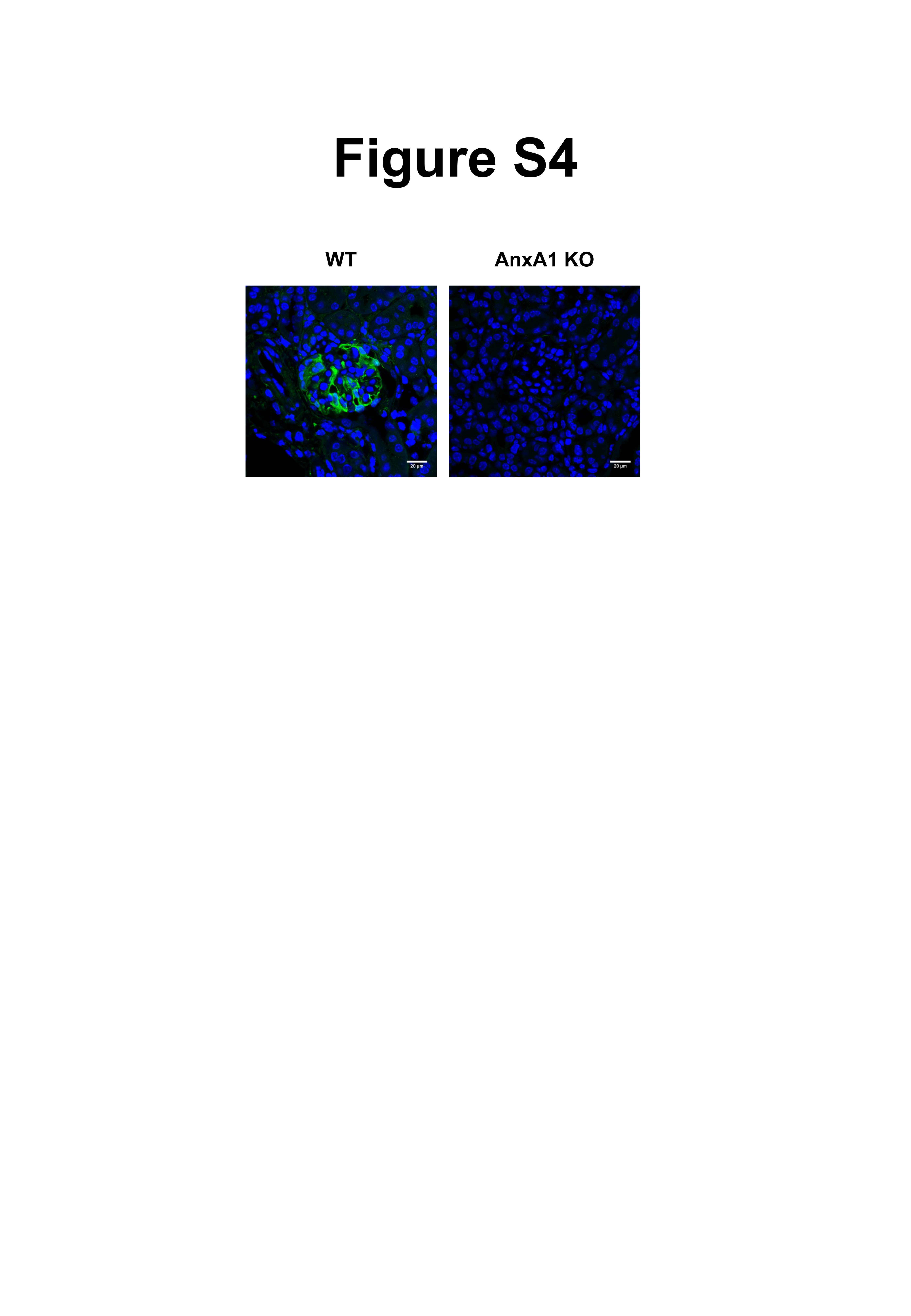

Supplement: Supplementary file 3 [file Image4.TIF]

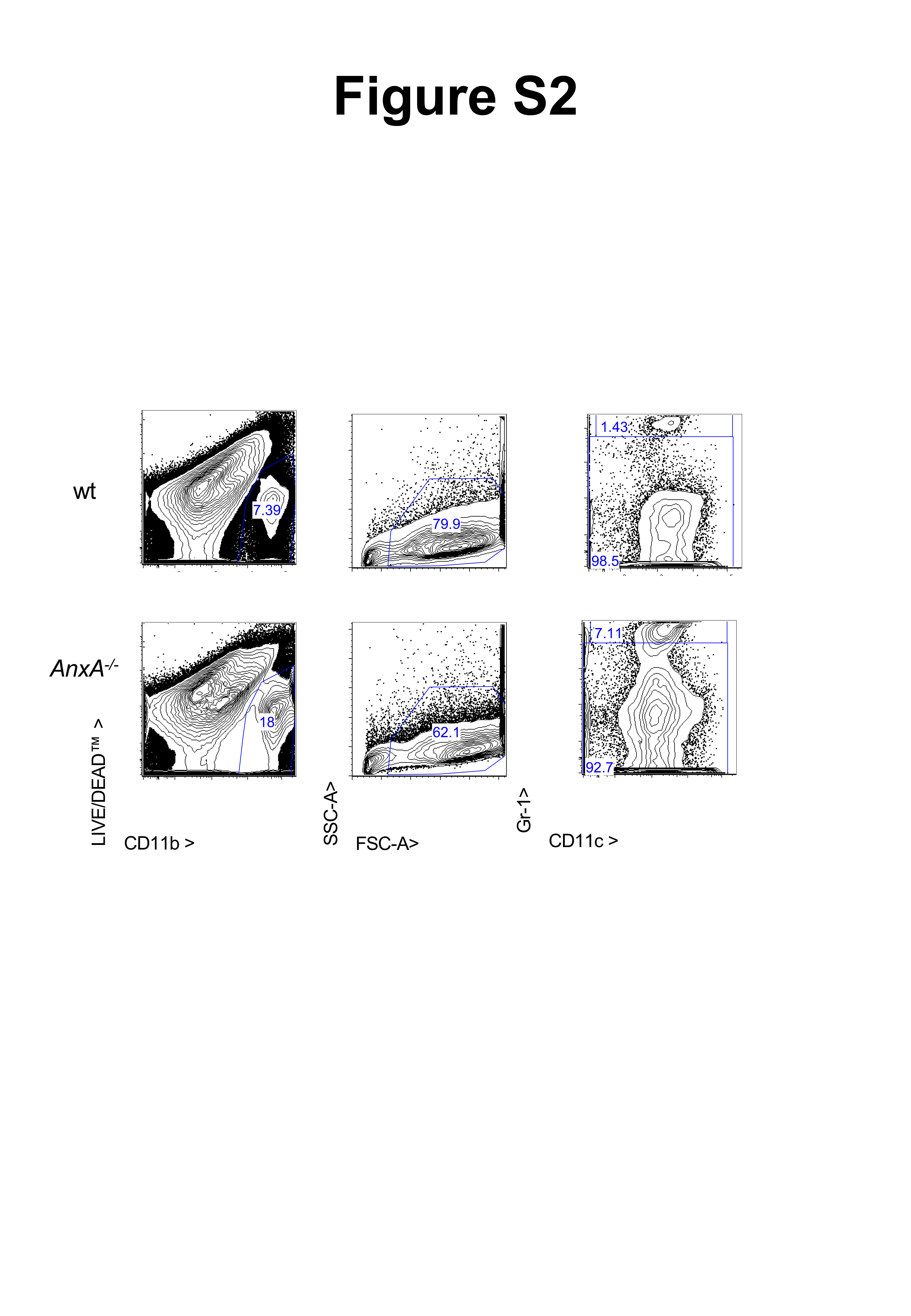

Supplement: Supplementary file 4 [file Image2.TIF]

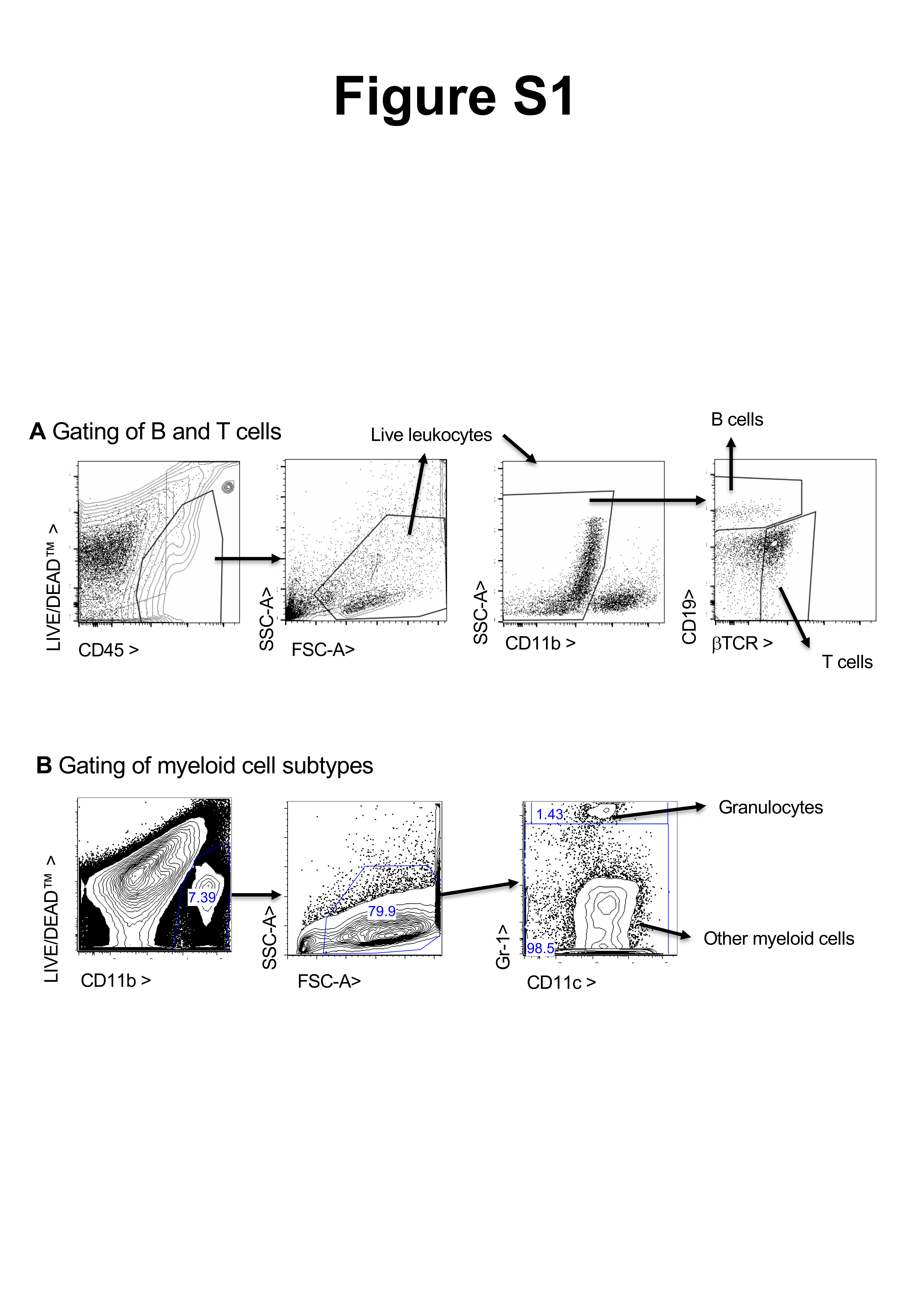

Supplement: Supplementary file 5 [file Image1.TIF]
