# Supplementary material for: Annexin A1 exerts renoprotective effects in experimental crescentic glomerulonephritis
Source: Front Physiol. 2022 Oct 12;13:984362. doi: 10.3389/fphys.2022.984362 (PMC9605209; doi:10.3389/fphys.2022.984362)
Supplement: Supplementary file 6 [file Table2.DOCX]

**Supplementary Table S2: List of lipids measured by mass-spectrometry**

|  | **WT NTN d10** | | **KO NTN d10** | |  |
| --- | --- | --- | --- | --- | --- |
| **Lipid** | **Mean**  **in ng/g** | **SD** | **Mean**  **in ng/g** | **SD** | ***t*-test** |
| 8,9-EEQ | 0.000 | 0.000 | 0.000 | 0.000 | - |
| 11,12-EEQ | 0.000 | 0.000 | 0.000 | 0.000 | - |
| 14,15-EEQ | 0.000 | 0.000 | 0.000 | 0.000 | - |
| 17,18-EEQ | 0.000 | 0.000 | 0.000 | 0.000 | - |
| 5,6-DiHETE | 1.268 | 0.852 | 0.284 | 0.491 | 0.079 |
| 8,9-DiHETE | 0.000 | 0.000 | 0.000 | 0.000 | - |
| 11,12-DiHETE | 0.000 | 0.000 | 0.000 | 0.000 | - |
| 14,15-DiHETE | 1.895 | 0.655 | 1.037 | 0.145 | 0.040 |
| 17,18-DiHETE | 4.361 | 1.341 | 3.511 | 0.574 | 0.294 |
| 5-HEPE | 0.653 | 0.244 | 0.420 | 0.141 | 0.136 |
| 8-HEPE | 0.223 | 0.176 | 0.142 | 0.036 | 0.427 |
| 9-HEPE | 0.086 | 0.228 | 0.000 | 0.000 | 0.506 |
| 12-HEPE | 93.686 | 164.781 | 24.060 | 15.548 | 0.459 |
| 15-HEPE | 1.303 | 0.608 | 1.496 | 0.336 | 0.601 |
| 18-HEPE | 4.087 | 1.838 | 3.388 | 1.103 | 0.537 |
| 19-HEPE | 0.000 | 0.000 | 0.000 | 0.000 | - |
| 20-HEPE | 0.000 | 0.000 | 0.000 | 0.000 | - |
| Resolvin E1 | 0.000 | 0.000 | 0.000 | 0.000 | - |
| LTB5 | 0.000 | 0.000 | 0.000 | 0.000 | - |
| 5,6-EET | 0.000 | 0.000 | 0.000 | 0.000 | - |
| 8,9-EET | 4.484 | 1.844 | 3.298 | 1.037 | 0.301 |
| 11,12-EET | 4.142 | 0.613 | 3.616 | 0.411 | 0.187 |
| 14,15-EET | 4.280 | 0.819 | 3.952 | 0.664 | 0.540 |
| 5,6-DHET | 2.259 | 0.271 | 2.652 | 0.293 | 0.062 |
| 8,9-DHET | 2.615 | 0.293 | 2.913 | 0.116 | 0.104 |
| 11,12-DHET | 2.002 | 0.276 | 2.309 | 0.224 | 0.109 |
| 14,15-DHET | 3.057 | 0.575 | 3.368 | 0.446 | 0.407 |
| 5-HETE | 9.646 | 2.742 | 10.963 | 1.330 | 0.426 |
| 8-HETE | 6.701 | 3.699 | 6.429 | 1.674 | 0.901 |
| 9-HETE | 6.968 | 1.803 | 7.141 | 1.826 | 0.889 |
| 11-HETE | 142.357 | 40.175 | 185.231 | 29.254 | 0.114 |
| 12-HETE | 544.005 | 677.044 | 290.729 | 154.924 | 0.515 |
| 15-HETE | 113.572 | 32.613 | 134.966 | 13.534 | 0.277 |
| 19-HETE | 0.000 | 0.000 | 0.000 | 0.000 | - |
| 20-HETE | 0.000 | 0.000 | 0.000 | 0.000 | - |
| LTB4 | 0.000 | 0.000 | 0.231 | 0.401 | 0.167 |
| LXA4 | 0.076 | 0.200 | 0.000 | 0.000 | 0.506 |
| 7,8-EDP | 2.512 | 0.744 | 1.708 | 0.239 | 0.083 |
| 10,11-EDP | 4.599 | 1.056 | 3.052 | 0.181 | 0.024 |
| 13,14-EDP | 2.964 | 0.600 | 1.970 | 0.280 | 0.017 |
| 16,17-EDP | 3.988 | 0.701 | 2.932 | 0.325 | 0.026 |
| 19,20-EDP | 12.926 | 1.258 | 10.810 | 1.205 | 0.029 |
| 7,8-DiHDPA | 0.000 | 0.000 | 0.000 | 0.000 | - |
| 10,11-DiHDPA | 0.243 | 0.060 | 0.266 | 0.039 | 0.541 |
| 13,14-DiHDPA | 0.670 | 0.121 | 0.602 | 0.107 | 0.401 |
| 16,17-DiHDPA | 9.165 | 3.272 | 6.284 | 1.092 | 0.149 |
| 19,20-DiHDPA | 12.108 | 3.330 | 8.155 | 1.740 | 0.069 |
| 4-HDHA | 6.533 | 1.750 | 5.709 | 0.490 | 0.419 |
| 7-HDHA | 2.175 | 0.984 | 1.336 | 0.355 | 0.162 |
| 8-HDHA | 5.447 | 1.586 | 4.121 | 0.706 | 0.176 |
| 11-HDHA | 6.699 | 2.666 | 5.173 | 1.255 | 0.345 |
| 10-HDHA | 6.389 | 5.419 | 4.555 | 1.514 | 0.558 |
| 13-HDHA | 18.009 | 7.901 | 12.945 | 4.060 | 0.297 |
| 14-HDHA | 97.451 | 147.478 | 51.913 | 29.596 | 0.589 |
| 16-HDHA | 8.456 | 3.092 | 6.737 | 2.070 | 0.380 |
| 17-HDHA | 18.952 | 8.547 | 22.900 | 8.174 | 0.501 |
| 20-HDHA | 27.120 | 8.756 | 19.638 | 6.232 | 0.194 |
| 21-HDHA | 0.000 | 0.000 | 0.000 | 0.000 | - |
| 22-HDHA | 0.000 | 0.000 | 0.000 | 0.000 | - |
| 10,17-DiHDHA | 0.000 | 0.000 | 0.000 | 0.000 | - |
| Resolvin D1 | 0.000 | 0.000 | 0.000 | 0.000 | - |
| 7,8-DiHDPA | 0.000 | 0.000 | 0.000 | 0.000 | - |
| 10,11-DiHDPA | 0.243 | 0.060 | 0.266 | 0.039 | 0.541 |
| 13,14-DiHDPA | 0.670 | 0.121 | 0.602 | 0.107 | 0.401 |
| 16,17-DiHDPA | 9.165 | 3.272 | 6.284 | 1.092 | 0.149 |
| 19,20-DiHDPA | 12.108 | 3.330 | 8.155 | 1.740 | 0.069 |
| 15-deoxy-delta 12.14-PGJ2 | 0.000 | 0.000 | 0.000 | 0.000 | - |
| PGJ2 | 0.000 | 0.000 | 0.000 | 0.000 | - |
| 2,3-dinor-6-keto-PGF1a | 0.000 | 0.000 | 0.000 | 0.000 | - |
| 15-keto-PGE2 | 0.858 | 0.460 | 2.570 | 1.778 | 0.042 |
| PGD2 | 35.704 | 9.391 | 63.535 | 27.585 | 0.041 |
| PGH2 | 0.000 | 0.000 | 0.000 | 0.000 | - |
| PGE2 | 202.656 | 78.798 | 485.691 | 249.759 | 0.023 |
| PGF2a | 214.433 | 52.839 | 276.808 | 117.813 | 0.275 |
| 11-dehydro TXB2 | 0.000 | 0.000 | 0.000 | 0.000 | - |
| TXB2 | 34.108 | 16.427 | 41.807 | 12.162 | 0.466 |
| 6-keto-PGF1a | 186.438 | 124.949 | 104.257 | 39.397 | 0.268 |
| PGE3 | 0.214 | 0.456 | 0.084 | 0.146 | 0.622 |
| d17-6-keto-PGF1a | 0.000 | 0.000 | 0.000 | 0.000 | - |
| 11-dehydro TXB3 | 0.000 | 0.000 | 0.000 | 0.000 | - |
| TXB3 | 0.000 | 0.000 | 0.000 | 0.000 | - |
| PGE1 | 0.000 | 0.000 | 0.000 | 0.000 | - |
| 13-HODE | 421.019 | 119.863 | 434.756 | 69.028 | 0.850 |
| 9,10-EpOME | 9.662 | 1.428 | 8.577 | 1.702 | 0.314 |
| 12,13-EpOME | 9.582 | 2.044 | 9.303 | 1.460 | 0.829 |
| 12,13-DiHOME | 15.515 | 2.791 | 19.563 | 3.137 | 0.065 |
| 9,10-DiHOME | 10.444 | 3.106 | 20.652 | 5.518 | 0.004 |
