# Supplementary material for: Annexin A1 exerts renoprotective effects in experimental crescentic glomerulonephritis
Source: Front Physiol. 2022 Oct 12;13:984362. doi: 10.3389/fphys.2022.984362 (PMC9605209; doi:10.3389/fphys.2022.984362)
Supplement: Supplementary file 7 [file Table3.DOCX]

**Supplementary Table S3: List of down-regulated genes sorted to enriched pathways**

| **Gene symbol** | **log2FoldChange** | **pvalue** |
| --- | --- | --- |
| **Electron Transport Chain** | | |
| Ndufa3 | -0.2662751 | 0.001952 |
| Ndufb10 | -0.29031343 | 0.00091976 |
| Ndufv3 | -0.24235213 | 0.00107066 |
| Ndufa12 | -0.37264832 | 9.9299E-07 |
| Cox6a1 | -0.21200609 | 0.00974953 |
| Ndufv1 | -0.26017788 | 0.00278135 |
| Atp5g2 | -0.33497712 | 1.6377E-07 |
| Cox7a1 | -0.37656432 | 0.0013271 |
| Atp5k | -0.26687386 | 0.00471425 |
| Cox11 | -0.28585002 | 0.00037818 |
| Atp5b | -0.25235857 | 0.0007247 |
| Ndufa5 | -0.26470897 | 0.00489551 |
| Ndufa1 | -0.25552111 | 0.00132814 |
| Atp5h | -0.23940313 | 0.00738955 |
| Ndufb6 | -0.23019535 | 0.00425974 |
| Ndufa9 | -0.19907647 | 0.00133508 |
| Atp5g1 | -0.29094817 | 5.4244E-05 |
| Cox5b | -0.25670371 | 8.0184E-05 |
| Ndufs5 | -0.22700197 | 0.00792083 |
| Atp5j | -0.27088305 | 0.0002986 |
| Ndufb3 | -0.32830422 | 7.6476E-05 |
| Uqcrc2 | -0.21619762 | 0.00294701 |
| Ndufs2 | -0.18867802 | 0.00566547 |
| Sdha | -0.24863827 | 0.00154935 |
| **TCA cycle** | | |
| Sucla2 | -0.22265364 | 0.00551338 |
| Dlst | -0.18806394 | 0.00824077 |
| Ogdh | -0.23572209 | 0.00102311 |
| Idh3b | -0.21014351 | 0.00491855 |
| Idh2 | -0.22898556 | 0.0067052 |
| Pdk2 | -0.20172203 | 0.00137148 |
| Pdhb | -0.20772907 | 0.00091626 |
| Pdhx | -0.18115799 | 0.0064026 |
| Idh3g | -0.24153435 | 0.00340238 |
| Dlat | -0.16674888 | 0.00374998 |
| Aco2 | -0.23189417 | 0.002201 |
| Mdh1 | -0.23899557 | 0.00373184 |
| Sdha | -0.24863827 | 0.00154935 |
| Aco1 | -0.21064474 | 0.00966853 |
| **Oxidative phosphorylation** | | |
| Ndufa3 | -0.2662751 | 0.001952 |
| Ndufb10 | -0.29031343 | 0.00091976 |
| Ndufv3 | -0.24235213 | 0.00107066 |
| Atp5h | -0.23940313 | 0.00738955 |
| Ndufb6 | -0.23019535 | 0.00425974 |
| Ndufa9 | -0.19907647 | 0.00133508 |
| Atp5g1 | -0.29094817 | 5.4244E-05 |
| Ndufs5 | -0.22700197 | 0.00792083 |
| Ndufv1 | -0.26017788 | 0.00278135 |
| Atp5j | -0.27088305 | 0.0002986 |
| Atp5g2 | -0.33497712 | 1.6377E-07 |
| Atp5k | -0.26687386 | 0.00471425 |
| Atp5b | -0.25235857 | 0.0007247 |
| Ndufs2 | -0.18867802 | 0.00566547 |
| Ndufa5 | -0.26470897 | 0.00489551 |
| Ndufa12 | -0.37264832 | 9.9299E-07 |
| Atp6v0c | -0.20634704 | 0.00041364 |
| Cox6a1 | -0.21200609 | 0.00974953 |
| Atp6v1b2 | -0.21174459 | 0.00194046 |
| Atp6v0a2 | -0.23269203 | 0.00589787 |
| Cox7a1 | -0.37656432 | 0.0013271 |
| Cox11 | -0.28585002 | 0.00037818 |
| Ndufa1 | -0.25552111 | 0.00132814 |
| Atp6v1g1 | -0.26089651 | 1.0951E-05 |
| Atp6v1g3 | -0.28976537 | 0.00401944 |
| Cox5b | -0.25670371 | 8.0184E-05 |
| Atp6v1c1 | -0.10455719 | 0.00726625 |
| Ndufb3 | -0.32830422 | 7.6476E-05 |
| Uqcrc2 | -0.21619762 | 0.00294701 |
| Ndufa13 | -0.26177116 | 0.00485798 |
| Sdha | -0.24863827 | 0.00154935 |
| **Amino Acid metabolism** | | |
| Ldha | -0.30149519 | 3.8685E-06 |
| Auh | -0.20766982 | 0.00314474 |
| Bhmt | -0.62728442 | 0.00229177 |
| Ass1 | -0.39785103 | 0.00629394 |
| Glul | -0.27153908 | 0.00433669 |
| Hmgcl | -0.22922679 | 0.00594717 |
| Hadh | -0.27242179 | 0.00651486 |
| Mdh1 | -0.23899557 | 0.00373184 |
| Got2 | -0.16405442 | 0.00240082 |
| Dlst | -0.18806394 | 0.00824077 |
| Ogdh | -0.23572209 | 0.00102311 |
| Mccc1 | -0.34824991 | 0.00564616 |
| Acadm | -0.41144675 | 7.8858E-06 |
| Adh5 | -0.26949372 | 0.00413295 |
| Pdhx | -0.18115799 | 0.0064026 |
| Prodh | -0.39672324 | 0.00029775 |
| Aco2 | -0.23189417 | 0.002201 |
| Sdha | -0.24863827 | 0.00154935 |
| **mRNA processing** | | |
| Dhx38 | -0.14941983 | 0.00125901 |
| Zrsr2 | -0.13330619 | 0.0031352 |
| Ppp1r8 | -0.19451685 | 0.00045383 |
| Eif4e2 | -0.13050107 | 0.00104921 |
| Rnmtl1 | -0.2471737 | 0.00483159 |
| Wdr55 | -0.20767312 | 0.00062886 |
| Aco1 | -0.21064474 | 0.00966853 |
| Zmat2 | -0.20359753 | 5.0065E-05 |
| Eif3b | -0.13756657 | 0.00294036 |
| Snrpd3 | -0.3453863 | 2.2363E-07 |
| Esrp2 | -0.12411319 | 0.00718772 |
| Rbmxl1 | -0.22931434 | 0.00347385 |
| Grsf1 | -0.20674536 | 0.00461618 |
| Park7 | -0.35781847 | 0.0001848 |
| Snrnp35 | -0.24955301 | 0.00336472 |
| Akap1 | -0.16253979 | 0.00756886 |
| Csad | -0.39163143 | 0.00021582 |
| Fbl | -0.2883987 | 5.7613E-06 |
| Rbm45 | -0.18653868 | 4.0005E-05 |
| Rae1 | -0.19535278 | 3.106E-05 |
| Slc6a8 | -0.15115305 | 0.00039606 |
| Auh | -0.20766982 | 0.00314474 |
| Rbm38 | -0.35198686 | 0.00052017 |
| Snrpb | -0.13765719 | 0.00299273 |
| Hnrnpab | -0.13483179 | 0.00221692 |
| Taf15 | -0.19647477 | 1.1158E-06 |
| U2af2 | -0.10731277 | 0.00027222 |
| Hnrnpa2b1 | -0.16461567 | 0.00078542 |
| Zfp622 | -0.1663794 | 0.00558834 |
| Ppm1g | -0.11643621 | 1.533E-06 |
| Rbm17 | -0.09767543 | 0.00756833 |
| Hnrnpll | -0.13476812 | 0.00312581 |
| Cfb | -0.63367424 | 6.5182E-05 |
| Clp1 | -0.21484088 | 0.00263107 |
| Srsf4 | -0.22904278 | 0.00013917 |
| **Glycolysis and Gluconeogenesis** | | |
| Ldha | -0.30149519 | 3.8685E-06 |
| Fbp1 | -0.23682278 | 0.00459055 |
| Got2 | -0.16405442 | 0.00240082 |
| Mpc1 | -0.39752609 | 0.00082449 |
| Pdhb | -0.20772907 | 0.00091626 |
| Pdhx | -0.18115799 | 0.0064026 |
| Gapdh | -0.37907849 | 1.5507E-05 |
| Aldob | -0.24680634 | 0.00704463 |
| Tpi1 | -0.21735993 | 0.00176977 |
| G6pc | -0.5561636 | 0.00134162 |
| Dlat | -0.16674888 | 0.00374998 |
| Mdh1 | -0.23899557 | 0.00373184 |
| **Proteasome Degradation** | | |
| Ubb | -0.34797456 | 0.00069827 |
| Psmb6 | -0.18667788 | 0.00689294 |
| Psmc1 | -0.17185816 | 0.0015347 |
| Psmb7 | -0.24830988 | 0.00034836 |
| Psmc3 | -0.15066961 | 0.00718937 |
| Psmb5 | -0.24927743 | 0.00014565 |
| H2afz | -0.25095739 | 4.1852E-05 |
| Psmd4 | -0.15446192 | 0.00467348 |
| Psmd2 | -0.13933873 | 0.00546435 |
| Psma6 | -0.22316413 | 0.00085915 |
| Psmd3 | -0.13584632 | 0.00232922 |
| Psmc4 | -0.19093357 | 0.00608639 |
| **Mitochondrial LC-Fatty Acid Beta-Oxidation** | | |
| Acadvl | -0.25891564 | 0.00025869 |
| Pecr | -0.31785947 | 0.00772868 |
| Hadha | -0.13564282 | 0.00068943 |
| Hadh | -0.27242179 | 0.00651486 |
| Acadm | -0.41144675 | 7.8858E-06 |
| Scp2 | -0.33730891 | 0.00124829 |
| **Synthesis and Degradation of Ketone Bodies** | | |
| Oxct1 | -0.34219328 | 4.7411E-05 |
| Bdh1 | -0.33390612 | 0.00096269 |
| Hmgcl | -0.22922679 | 0.00594717 |
| Acat1 | -0.46608821 | 0.00184815 |
| **Nuclear Receptors** | | |
| Esrrb | -0.19227815 | 0.00491639 |
| Rorc | -0.77424106 | 0.00018401 |
| Nr1h3 | -0.29197406 | 0.00230668 |
| Ar | -0.26657991 | 0.00116362 |
| Ppard | -0.59518375 | 0.00060817 |
| Hnf4a | -0.35618116 | 0.00347444 |
| Esrra | -0.26673922 | 0.00771288 |
| Rxrb | -0.20101408 | 0.00177448 |
