# Supplementary material for: Annexin A1 exerts renoprotective effects in experimental crescentic glomerulonephritis
Source: Front Physiol. 2022 Oct 12;13:984362. doi: 10.3389/fphys.2022.984362 (PMC9605209; doi:10.3389/fphys.2022.984362)
Supplement: Supplementary file 8 [file Table4.DOCX]

**Supplementary Table S4: List of up-regulated genes sorted to enriched pathways**

| **Gene symbol** | **log2FoldChange** | **pvalue** |
| --- | --- | --- |
| **B Cell Receptor Signaling Pathway** | | |
| Rela | 0.241311411 | 4.28E-03 |
| Zap70 | 0.725531941 | 1.13E-04 |
| Ptprc | 0.550693999 | 9.57E-03 |
| Blnk | 0.32253138 | 4.99E-03 |
| Pik3r1 | 0.211256847 | 1.80E-03 |
| Lcp2 | 0.708231632 | 6.28E-04 |
| Pik3ap1 | 0.641608511 | 6.29E-03 |
| Syk | 0.633861198 | 3.78E-03 |
| Ccnd2 | 0.287555741 | 3.50E-03 |
| Rel | 0.582288606 | 8.94E-04 |
| Hck | 0.68380906 | 2.75E-03 |
| Ptpn18 | 0.368364229 | 7.03E-04 |
| Rps6ka1 | 0.316798993 | 5.43E-03 |
| Pip5k1b | 0.371448412 | 2.08E-06 |
| Vav1 | 0.597234802 | 7.69E-03 |
| Pik3cg | 0.64619643 | 2.72E-03 |
| Bank1 | 0.699131785 | 4.90E-03 |
| Lyn | 0.328549204 | 3.47E-03 |
| Rb1 | 0.320246751 | 4.90E-05 |
| Ptpn6 | 0.216442602 | 1.14E-03 |
| Hdac7 | 0.259221956 | 7.55E-03 |
| Ppp3ca | 0.196904161 | 1.77E-04 |
| Rac3 | 0.315176632 | 8.96E-03 |
| Rela | 0.241311411 | 4.28E-03 |
| Pik3r5 | 0.663633447 | 6.61E-03 |
| Vav3 | 0.390425339 | 2.72E-03 |
| Kras | 0.138107242 | 3.49E-03 |
| Pirb | 0.756740619 | 1.03E-03 |
| Rac2 | 0.656543837 | 4.43E-03 |
| **Toll-like receptor signaling pathway** | | |
| Rela | 0.241311411 | 4.28E-03 |
| Tlr3 | 0.385920755 | 7.64E-04 |
| Pik3r5 | 0.663633447 | 6.61E-03 |
| Mapk11 | 0.502864961 | 1.61E-03 |
| Ccl3 | 0.844106302 | 7.30E-04 |
| Pik3r1 | 0.211256847 | 1.80E-03 |
| Tlr5 | 0.649357505 | 9.79E-03 |
| Il6 | 0.951043173 | 3.00E-04 |
| Il1b | 0.832393822 | 2.47E-04 |
| Lbp | 0.659457873 | 2.55E-03 |
| Ifnar1 | 0.165017037 | 7.00E-03 |
| Tlr1 | 1.01167362 | 2.01E-06 |
| Tlr7 | 0.48846831 | 2.51E-03 |
| Tlr6 | 0.763502947 | 2.86E-04 |
| Cd40 | 0.589087161 | 2.66E-03 |
| Pik3cg | 0.64619643 | 2.72E-03 |
| Cxcl9 | 0.57707867 | 2.49E-03 |
| **EGFR1 Signaling Pathway** | | |
| Sh3bgrl | 0.383518906 | 3.63E-04 |
| Cebpb | 0.314776695 | 8.30E-03 |
| Pik3r1 | 0.211256847 | 1.80E-03 |
| Vav3 | 0.390425339 | 2.72E-03 |
| Kras | 0.138107242 | 3.49E-03 |
| Tgif1 | 0.448008114 | 3.65E-03 |
| Tnip1 | 0.213128116 | 1.71E-03 |
| Dok2 | 0.485387433 | 3.72E-03 |
| Dnm1 | 0.468729107 | 3.84E-04 |
| Grb14 | 0.225119475 | 8.48E-03 |
| Cav1 | 0.279763288 | 8.71E-04 |
| Klf11 | 0.571248499 | 4.19E-05 |
| Rps6ka1 | 0.316798993 | 5.43E-03 |
| Ralgds | 0.195049213 | 4.31E-03 |
| Map3k14 | 0.502052587 | 6.25E-08 |
| Socs3 | 0.780609155 | 3.31E-03 |
| Cav2 | 0.27720502 | 3.91E-03 |
| Pik3cg | 0.64619643 | 2.72E-03 |
| Map3k1 | 0.391759492 | 9.02E-03 |
| Vav1 | 0.597234802 | 7.69E-03 |
| **Complement and Coagulation Cascades** | | |
| Kng1 | 0.987006238 | 5.57E-05 |
| C3ar1 | 0.681723485 | 9.81E-03 |
| Cfh | 0.478636083 | 2.67E-03 |
| Plat | 0.412466847 | 1.52E-04 |
| Thbd | 0.384705209 | 3.54E-04 |
| C1s1 | 0.474495869 | 7.59E-03 |
| Pros1 | 0.450768762 | 7.83E-03 |
| F7 | 1.061384134 | 6.88E-05 |
| Serping1 | 0.547859282 | 3.93E-03 |
| Tfpi | 0.399279827 | 4.28E-03 |
| Cd46 | 0.514709018 | 7.88E-04 |
| F10 | 0.731593251 | 3.33E-03 |
| C6 | 0.820486181 | 1.61E-03 |
| Serpina1a | 0.490251814 | 6.20E-03 |
| Serpina1b | 0.815175623 | 1.47E-03 |
| Kng2 | 0.175883893 | 2.91E-03 |
| Bdkrb2 | 0.976760775 | 6.17E-05 |
| **T Cell Receptor Signaling Pathway** | | |
| Cebpb | 0.314776695 | 8.30E-03 |
| Zap70 | 0.725531941 | 1.13E-04 |
| Wasf2 | 0.138685099 | 9.53E-03 |
| Ptprc | 0.550693999 | 9.57E-03 |
| Pik3r1 | 0.211256847 | 1.80E-03 |
| Lcp2 | 0.708231632 | 6.28E-04 |
| Vav3 | 0.390425339 | 2.72E-03 |
| Arhgdib | 0.387920212 | 8.80E-03 |
| Syk | 0.633861198 | 3.78E-03 |
| Dtx1 | 0.537855689 | 2.45E-03 |
| Fyb | 0.669924211 | 6.98E-04 |
| Sh2b3 | 0.198489193 | 1.48E-03 |
| Map3k1 | 0.391759492 | 9.02E-03 |
| Vav1 | 0.597234802 | 7.69E-03 |
| Lyn | 0.328549204 | 3.47E-03 |
| Rac2 | 0.656543837 | 4.43E-03 |
| Ptpn6 | 0.216442602 | 1.14E-03 |
| Hdac7 | 0.259221956 | 7.55E-03 |
| **Apoptosis** | | |
| Rela | 0.241311411 | 4.28E-03 |
| Tnfrsf10b | 0.479457274 | 2.35E-05 |
| Casp1 | 0.595983139 | 1.63E-03 |
| Pik3r1 | 0.211256847 | 1.80E-03 |
| Irf1 | 0.453062522 | 2.25E-03 |
| Lta | 0.853291729 | 1.28E-03 |
| Traf1 | 0.722604665 | 2.01E-04 |
| Tnfrsf1b | 0.675217008 | 1.72E-04 |
| Trp63 | 0.72505613 | 8.55E-07 |
| Casp4 | 0.672876014 | 7.35E-03 |
| Map3k1 | 0.391759492 | 9.02E-03 |
| Igf1 | 0.565332493 | 2.95E-03 |
| Mcl1 | 0.150837154 | 6.27E-03 |
| Casp2 | 0.223340655 | 1.06E-03 |
| **Kit Receptor Signaling Pathway** | | |
| Tnfrsf10b | 0.479457274 | 2.35E-05 |
| Matk | 0.596559983 | 5.18E-03 |
| Pik3r1 | 0.211256847 | 1.80E-03 |
| Plce1 | 0.303886046 | 1.42E-04 |
| Fes | 0.489617972 | 2.43E-03 |
| Hck | 0.68380906 | 2.75E-03 |
| Rps6ka1 | 0.316798993 | 5.43E-03 |
| Pik3cg | 0.64619643 | 2.72E-03 |
| Vav1 | 0.597234802 | 7.69E-03 |
| Lyn | 0.328549204 | 3.47E-03 |
| Ptpn6 | 0.216442602 | 1.14E-03 |
| Socs1 | 0.682059573 | 9.72E-06 |
| **Chemokine signaling pathway** | | |
| Rela | 0.241311411 | 4.28E-03 |
| Xcr1 | 0.592022058 | 1.71E-03 |
| Pik3r5 | 0.663633447 | 6.61E-03 |
| Ccl3 | 0.844106302 | 7.30E-04 |
| Pik3r1 | 0.211256847 | 1.80E-03 |
| Vav3 | 0.390425339 | 2.72E-03 |
| Gng8 | 0.60832283 | 7.09E-03 |
| Kras | 0.138107242 | 3.49E-03 |
| Cx3cl1 | 0.484592434 | 6.17E-04 |
| Hck | 0.68380906 | 2.75E-03 |
| Ccl1 | 0.785670103 | 1.58E-03 |
| Vav1 | 0.597234802 | 7.69E-03 |
| Pik3cg | 0.64619643 | 2.72E-03 |
| Gng7 | 0.534869912 | 8.09E-04 |
| Ccl8 | 0.85071486 | 8.26E-04 |
| Lyn | 0.328549204 | 3.47E-03 |
| Rac2 | 0.656543837 | 4.43E-03 |
| Cxcl9 | 0.57707867 | 2.49E-03 |
| **IL-2 Signaling Pathway** | | |
| Rela | 0.241311411 | 4.28E-03 |
| Ets2 | 0.207927168 | 2.65E-05 |
| Pik3r1 | 0.211256847 | 1.80E-03 |
| Syk | 0.633861198 | 3.78E-03 |
| Kras | 0.138107242 | 3.49E-03 |
| Cd53 | 0.596276994 | 7.39E-03 |
| Socs3 | 0.780609155 | 3.31E-03 |
| Pik3cg | 0.64619643 | 2.72E-03 |
| Vav1 | 0.597234802 | 7.69E-03 |
| Lyn | 0.328549204 | 3.47E-03 |
| Ptpn6 | 0.216442602 | 1.14E-03 |
| Socs1 | 0.682059573 | 9.72E-06 |
| Il2rg | 0.669738136 | 2.34E-04 |
